# Neural correlates of sparse coding and dimensionality reduction

**DOI:** 10.1371/journal.pcbi.1006908

**Published:** 2019-06-27

**Authors:** Michael Beyeler, Emily L. Rounds, Kristofor D. Carlson, Nikil Dutt, Jeffrey L. Krichmar

**Affiliations:** 1 Department of Psychology, University of Washington, Seattle, Washington, United States of America; 2 Institute for Neuroengineering, University of Washington, Seattle, Washington, United States of America; 3 eScience Institute, University of Washington, Seattle, Washington, United States of America; 4 Department of Computer Science, University of California, Irvine, California, United States of America; 5 Department of Cognitive Sciences, University of California, Irvine, California, United States of America; 6 Sandia National Laboratories, Albuquerque, New Mexico, United States of America; Imperial College London, UNITED KINGDOM

## Abstract

Supported by recent computational studies, there is increasing evidence that a wide range of neuronal responses can be understood as an emergent property of nonnegative sparse coding (NSC), an efficient population coding scheme based on dimensionality reduction and sparsity constraints. We review evidence that NSC might be employed by sensory areas to efficiently encode external stimulus spaces, by some associative areas to conjunctively represent multiple behaviorally relevant variables, and possibly by the basal ganglia to coordinate movement. In addition, NSC might provide a useful theoretical framework under which to understand the often complex and nonintuitive response properties of neurons in other brain areas. Although NSC might not apply to all brain areas (for example, motor or executive function areas) the success of NSC-based models, especially in sensory areas, warrants further investigation for neural correlates in other regions.

## Author summary

Brains face the fundamental challenge of extracting relevant information from high-dimensional external stimuli in order to form the neural basis that can guide an organism's behavior and its interaction with the world. One potential approach to addressing this challenge is to reduce the number of variables required to represent a particular input space (i.e., dimensionality reduction). We review compelling evidence that a range of neuronal responses can be understood as an emergent property of nonnegative sparse coding (NSC)—a form of efficient population coding due to dimensionality reduction and sparsity constraints.

## Introduction

Brains face the fundamental challenge of extracting relevant information from high-dimensional external stimuli in order to form the neural basis that can guide an organism's behavior and its interaction with the world. To support complex patterns of behavior, populations of interconnected neurons must implement a rich repertoire of linear and nonlinear operations on their synaptic inputs that take into account context, experience, and anatomical constraints [1]. For example, anatomical bottlenecks often force the information stored in a large number of neurons to be compressed into an orders-of-magnitude-smaller population of downstream neurons [2–4], such as storing information from 100 million photoreceptors in 1 million optic nerve fibers or resulting in a 10–10,000-fold convergence from cortex to the basal ganglia [3].

**Fig 1:**
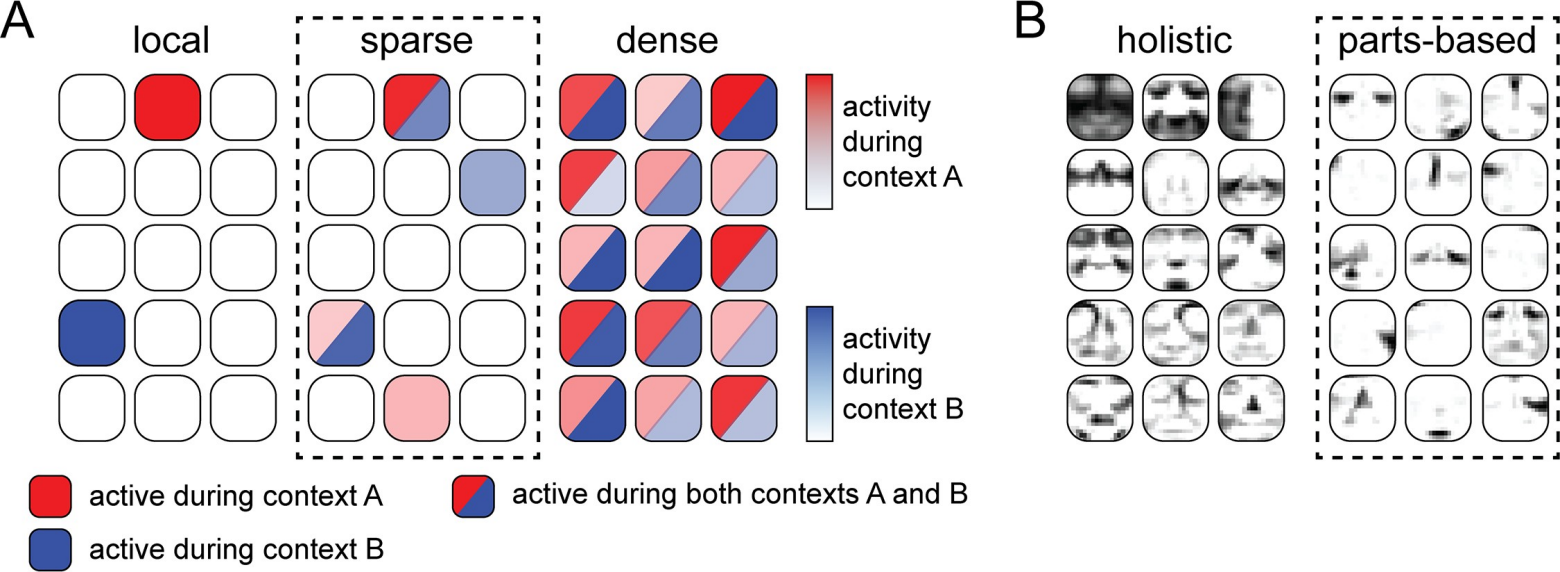
NSC promotes population codes that are both sparse and parts based. (A) Hypothetical activity in a population of neurons during presentation of two different external stimuli (“contexts”). A sparse code is a trade-off between a local code (in which a context is represented by the activity of a single neuron, and different contexts are represented by different neurons) and a dense code (in which all neurons are active, and their combined activity is used to encode each context). Dense codes possess great memory capacity but suffer from cross talk among neurons, whereas local codes do not suffer from interference but also have no capacity for generalization (inspired by [8]). (B) In a holistic representation of faces, individual neurons in the population respond themselves to faces as a whole [11], whereas in a parts-based representation, individual neurons explicitly encode individual face components [12], such as the eyes, nose, and mouth (inspired by [13]). NSC, nonnegative sparse coding.

One potential approach to addressing this challenge is to reduce the number of signals required to transmit information in the network—for example, through **sparse-coding** schemes (text in bold appear in the Glossary section), in which information is represented by the activity of a small proportion of neurons in a population [5–7]. A number of different definitions of sparsity can be found in the literature [8, 9], which can sometimes lead to controversy as to which codes can still be considered sparse [8]. An extreme example is the so-called local code, in which each unique event, or “context,” is encoded by a single active neuron, or “grandmother cell” [10] (illustrated in the left column of Fig 1A). Local codes not only suffer from low **representational capacity**, because they allow a population of *N* neurons to encode at most *N* contexts, but also require a large number of neurons to cover the space of possible contexts. On the other hand, a dense code represents each context by the combined activity of all neurons in the population (Fig 1A, right column). In theory, dense codes lead to high representational capacity (at *M* activity levels, allowing for *M*^*N*^ contexts to be encoded), but they also suffer from neuronal cross talk because every neuron is involved in every context. Alternatively, sparse codes (Fig 1A, center column) can be described as a trade-off between the benefits and drawbacks of dense and local codes, in which each context is encoded by a different subset of neurons in the population. [5]. In general, sparse coding reduces the overall neural activity necessary to represent information.

Another approach to address this challenge is to reduce the number of variables required to represent a particular input, stimulus, or task space, a process known as **dimensionality reduction**. Although responses of individual neurons are often complex and highly nonlinear, a population of neurons might share activity patterns because of individual neurons in the population not being independent of each other. Dimensionality reduction methods have proved useful in elucidating these shared activity patterns and thus effectively explaining population activity using a lower number of variables than there are neurons in the population (for a recent review, see [14]).

Neurons often encode several behaviorally relevant variables simultaneously [15–18], allowing for multifaceted representations of high-dimensional stimulus spaces. For example, a population of neurons tasked with encoding human faces might opt to represent each individual face as a combination of a set of standard faces (Fig 1B, left column). In such a **holistic representation** of faces [11], each individual neuron would itself respond to a face as a whole (i.e., a face “template”) without explicitly representing individual face components, and an arbitrary face could be represented by combining different face templates (e.g., by adding 10% of template 1 to 20% of template 2 and subtracting 30% of template 3). On the other hand, faces can also be represented as a combination of individual face components, such as eyes, noses, and mouth, in what is known as a **parts-based representation** (Fig 1B, right column) [12, 19]. Both approaches allow for representing arbitrary faces as a combination of neural activity but have drastically different consequences on the set of stimulus features each neuron responds to. Although visual information from the eyes, nose, and mouth would of course be included in a holistic face representation, that information would not be explicitly represented as structural units in their own right [11]. Linear combinations of holistic components often involve complex cancellations between positive and negative contributions and thus lack the intuitive meaning of adding parts to form a whole. In contrast, a parts-based representation allows for only nonsubtractive combinations of stimulus features [12]. Although the relevant stimulus dimensions are often not known a priori, several sophisticated mathematical techniques exist that allow us to discover these representations directly from experimental data [14, 19–23].

In this article, we review evidence from experimental and theoretical studies suggesting that a number of neuronal responses can be understood as an emergent property of nonnegative sparse coding (NSC), an efficient population coding scheme based on dimensionality reduction and sparsity constraints. In particular, we review evidence for NSC in sensory areas that efficiently encode external stimulus spaces, for associative areas to conjunctively represent multiple behaviorally relevant variables, and for the basal ganglia to coordinate movement.

## Nonnegative sparse coding as a modern variant of the efficient coding hypothesis

### Efficient coding

The fundamental principle of **efficient coding** is that a sensory system is adjusted to the specific statistics of the natural environment from which it encodes and transmits information [24–27]. Efficiency, in this context, is an information-theoretic term that should not be confused with “minimizing energy expenditure.” Instead, a sensory pathway is treated as a noisy communication channel, in which the goal is to maximize the rate at which information can be reliably transmitted by minimizing the redundancy between representational units.

**Fig 2:**
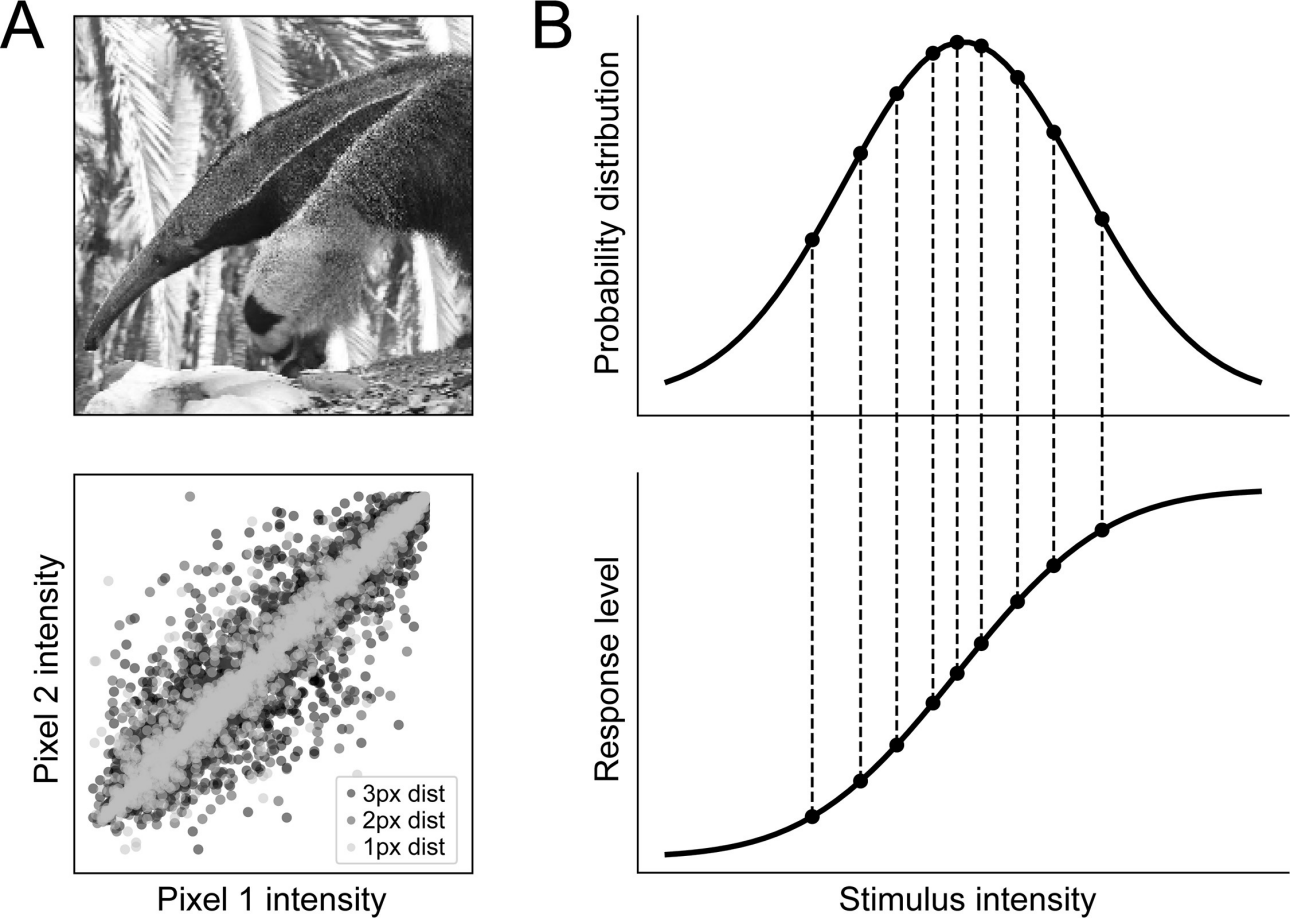
Efficient coding hypothesis. (A) Sensory stimuli in the environment, such as an image of an anteater, display significant statistical structure. For example, the luminance value of nearby pixels in the image is significantly correlated, an effect that exists even for nonadjacent pixels (inspired by [27]). Neural systems can improve their coding efficiency by accounting for and reducing such information redundancy. (B) For a given distribution of sensory characteristics in the world (top), a neuron's information capacity is maximized when all response levels are used with equal frequency (inspired by [29]). Intervals between each response level encompass an equal area under the intensity distribution, so each state is used with equal frequency.

Early theories of efficient coding [24, 25] were developed based on the visual system. Attneave [25] pointed out that there is a significant degree of redundancy in natural visual images because of correlations in both the spatial and temporal domains (for a recent review, see [28]). For example, the luminance values of a pair of pixels separated by a fixed distance in a natural image are likely to be highly correlated (Fig 2A). These statistical regularities constrain the images a visual system is likely to encounter to a tiny fraction of the set of all possible images. It was therefore argued that the visual system should not waste resources on processing arbitrary images but instead use statistical knowledge about its environment to represent the relevant input space as economically as possible.

Extending this idea to the neural level, Barlow [24] proposed that the goal of early neurons in sensory processing is to transform raw visual inputs into an efficient representation such that as much information as possible can be extracted from them given limited neural resources. This efficient coding principle has been able to explain a wide variety of neuronal response properties in the early visual system, such as the center-surround structure of receptive fields (RFs) in the retina [30], temporally decorrelated signals in the lateral geniculate nucleus (LGN) [31], and the coding of natural scenes in the primary visual cortex (V1) [9].

At the level of single neurons, efficient coding suggests that the information carried by a neuron's response can be maximized by using all response levels with equal frequency [29, 32, 33]. For example, in the case of a neuron representing a single input variable with a single output variable, information is maximized when the input–output function corresponds to the cumulative probability function for the different input levels [29], as shown in Fig 2B. Note that this coding procedure amplifies inputs in proportion to their expected frequency of occurrence rather than reserving large portions of its dynamic range for improbable inputs [29, 32]. On the other hand, if the input–output function sensitivity is chosen as too low, high levels of the stimulus feature will be indistinguishable as the response function saturates; if the sensitivity is set too high, low levels of the stimulus feature cannot drive responses [29].

At the level of neuronal populations, neural responses should be both decorrelated (i.e., independent from one another) and sparse (i.e., involve only a small fraction of neurons in the population) [27].

### Sparse coding

Taking these ideas a step further, Olshausen and Field [34] noted that natural images contain statistical dependencies beyond linear pairwise correlations among image pixels and argued that these higher-order correlations should be taken into account when developing an efficient code. Their goal was thus to find a linear coding strategy capable of reducing these higher-order forms of redundancy.

Linear sparse coding is one such strategy, in which monochromatic images *I*(*x*,*y*) are described in terms of a linear superposition of a number of *B* **basis functions**, *w*_*b*_(*x*,*y*):

$$I(x,y)=\sum_{b=1}^{B}w_{b}(x,y)h_{b},$$ (1)

where *h*_*b*_ are stochastic coefficients that are different for each image [35, 36]. Learning a sparse code for images thus involved determining the values of both *w*_*b*_(*x*,*y*) and *h*_*b*_ for all *b* and (*x*,*y*), given a sufficient number of observation of images, under the constraint that *h*_*b*_ be sparse. In this context, *h*_*b*_ was considered sparse if it took very small or very large (absolute) values more often than a Gaussian random variable would [36]. This sparsity constraint allowed for basis functions that were not needed to describe a given image structure to be weeded out.

When Olshausen and Field applied linear sparse coding to natural images, they found that the emerging basis functions were qualitatively similar in form to RFs of simple cells in V1 [35, 37], thus giving empirically observed RFs an information-theoretic explanation. In this context, *h*_*b*_ in Eq 1 corresponded to the (signed) activation value of a particular V1 neuron, and *w*_*b*_(*x*,*y*) were the connection weights (or synaptic weights in an artificial neural network) that were closely related to that neuron's RF.

Sparsity, in this context, is an information-theoretic concept related to how efficiently and completely information is encoded with the basis functions described previously. Please note that this is different from empirical observations of brain areas being “sparsely” activated; that is, sparse population activity does not necessarily imply that a brain area implements a sparse-coding scheme. This confusion is fueled in part by the wide variety of definitions of sparsity used in the literature [8, 38]. For example, even though sparse coding (as a theoretical framework) applied to natural images yields V1-like RFs, recent evidence suggests that neural activity in V1 might not be as sparsely activated as previously thought [39, 40]. However, V1 still codes stimuli efficiently [40].

Olshausen and Field went on to show that the set of basis functions that best described V1 RFs was greater in number than the effective dimensionality of the input (which they termed an overcomplete basis set) [37]. It is worth noting that sparse coding with an overcomplete basis set is typically associated with an anatomical fan-out motif, such as expanding 1 million optic nerve fibers into more than 100 million V1 neurons or from a small number of mossy fibers to a 100-fold–larger number of granule cells in the cerebellum.

However, as pointed out by Hoyer [41], linear sparse coding falls short of providing a literal interpretation for V1 simple-cell behavior for two reasons: (1) every neuron could be either positively or negatively active, and (2) the input to the neural network was typically double-signed, whereas V1 neurons receive visual input from the LGN in the form of separated, nonnegative ON and OFF channels.

In order to transform Olshausen and Field's sparse coding from a relatively abstract model of image representation into a biologically plausible model of early visual cortex processing, Hoyer [41, 42] thus proposed to enforce both input signal and neuronal activation to be nonnegative (though still allowing inhibitory connections). This seemingly simple change had remarkable consequences on the quality of the sensory representation: whereas elementary image features in the standard sparse-coding model could “cancel each other out” through subtractive interactions, enforcing nonnegativity ensured that features combined additively, much like the intuitive notion of combining parts to form a whole. The resulting parts-based representations resembled RFs in V1 much more closely than other holistic representations. These considerations led to the formulation of NSC in its current form.

### Nonnegative sparse coding

As a special case of linear sparse coding, NSC shares the same goal of accurately describing observed data as a superposition of a set of sparsely activated basis functions, as well as enforcing dimensionality reduction. In addition, NSC requires all basis functions and activation values (i.e., *w*_*b*_(*x*,*y*) and *h*_*b*_ in Eq 1) to be nonnegative. However, NSC is more than just linear sparse coding with nonnegative weights. For example, whereas linear sparse coding typically uses a larger number of basis functions than there are dimensions in the input (thus achieving dimensionality expansion), NSC makes use of **nonnegative matrix factorization (NMF)** to achieve dimensionality reduction. This has interesting implications for the kinds of basis functions that can be learned. Most prominently, the nonnegativity constraints used in NMF force the different basis functions to add up linearly, thus leading to the distinctive parts-based representations.

Consider *S* observed stimuli or data samples, each composed of *F* observed feature values, such as a collection of *S* images *I*(*x*,*y*)_*s*_ (*s*∈[1,…,*S*]) from the previous example, each consisting of *F* different grayscale values. If we arrange the observed feature values of the *s*-th observation into a vector ${\vec{v}}_{s}$ (i.e., by flattening each observed image), and if we arrange all vectors into the columns of an *F*×*S* data matrix **V**, then linear decompositions describe these data as

V≈WH, (2)

where **W** is an *F*×*B* matrix that contains as its columns the *B* basis functions of the decomposition (i.e., the *b*-th column of **W** corresponding to *w*_*b*_(*x*,*y*) ∀*x*,*y* in Eq 1), and **H** is a *B*×*S* matrix containing as its columns the activation values of each basis function for a particular input stimulus (i.e., the *b*-th column of **H** corresponding to *h*_*b*_ ∀*b* in Eq 1). The difference between **V** and **WH** is termed the reconstruction error.

The goal of NSC is then to find a linear decomposition of **V** that minimizes the reconstruction error while guaranteeing that **H** is sparse. This can be achieved by minimizing the following cost function [42]:

$$\underset{W,H}{\min}\frac{1}{2}\|V-\mathrm{WH}{\|}^{2}+\lambda \sum_{ij}f(H_{ij}),$$ (3)

subject to the constraints ∀*ij*:**W**_*ij*_≥0, **H**_*ij*_≥0, and $\|{\vec{w}}_{i}\|=1$, where ${\vec{w}}_{i}$ denotes the *i*-th column of **W**. Here, the left-hand term describes the reconstruction error, whereas the right-hand term describes the sparsity of the decomposition. The trade-off between accurate reconstruction and sparsity is controlled by the parameter *λ* (where *λ*≥0), whereas the form of *f* defines how sparsity is measured (a typical choice is the L1 norm on **H**).

Analogous to efficient coding, Eq 3 forces prediction errors to be amplified in proportion to their expected frequency of occurrence because a more frequent event would show up more frequently in **V**. Hence, accounting for a rare observation at the expense of ignoring a more common one would result in an increased reconstruction error.

In the case of *λ* = 0, Eq 3 reduces to the squared-error version of NMF. Although NMF enforces all elements of **W** and **H** to be nonnegative, the resulting decomposition might not be sparse, depending on the number of basis functions *B*. In order to emphasize decompositions in which **H** is sparse, Eq 3 should be minimized with *λ*>0 [42].

**Fig 3:**
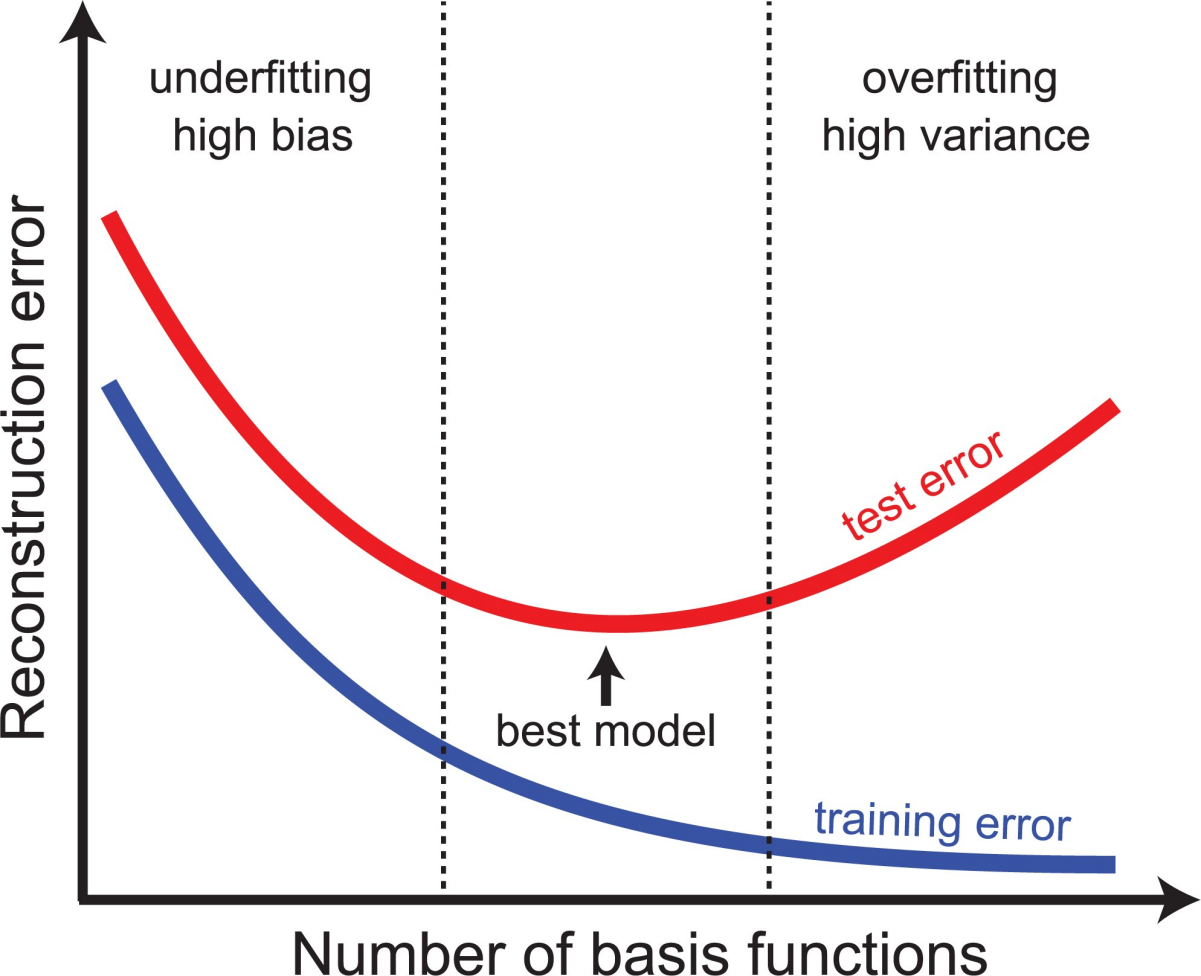
The bias–variance dilemma. With increased model complexity (i.e., with an increased number of basis functions), the reconstruction error on a set of familiar (training) data typically decreases until it reaches zero. In contrast, the reconstruction error on a set of unfamiliar, held-out (test) data typically goes through a minimum as a function of model complexity. A successful model chooses the number of basis functions such that the generalization (test) error is minimized (labeled “best model”).

Another open parameter is the number of basis functions, *B*, which controls the predictive power of the model and must be determined empirically. With a small number of basis functions, NSC is unlikely to achieve a low reconstruction error, be it in familiar contexts (training data) or in novel contexts (held-out test data). In this case, the error depends on the systematic bias of the model, and the model is said to underfit the data (left-hand side of Fig 3). With increased model complexity, the model can learn subtle differences between different contexts with high accuracy, leading to a reduced bias (training) error. However, with increased complexity, the model is more likely to learn patterns between training contexts that arise either from underlying noise or from spurious correlations. As a result, the model will respond according to these learned patterns when a novel context is presented (rather than according to the underlying actual relationships), in which case the model is said to overfit the data (right-hand side of Fig 3). Hence, the goal of a successful model is to find the ideal compromise in the bias–variance error trade-off [43] (labeled “best model” in Fig 3).

Analogously to [35, 37], the basis functions obtained in NSC can be interpreted as the connection weights of a population of simulated neurons in an artificial neural network. In other words, under NSC, the number of basis functions *B* corresponds to the number of output neurons, and the response of the *b*-th model output neuron (*b*∈[1,…,*B*]) to a particular input stimulus *s*, termed *r*_*bs*_, can be computed by feeding the dot product of that neuron's connection weights (i.e., the *b*-th column in **W**, ${\vec{w}}_{b}$) and a data vector (i.e., the *s*-th column in **V**, ${\vec{v}}_{s}$) to an activation function Θ:

$$r_{bs}=\Theta ({\vec{w}}_{b}\cdot {\vec{v}}_{s}),$$ (4)

where “⋅” denotes the dot product. For example, the linear response of a model neuron can be calculated by setting Θ to the identity function Θ(*x*) = *x*. Note that the response of the model neuron to different stimuli *s*∈[1,…,*S*] involves different columns of **V** but always relies on ${\vec{w}}_{b}$.

Thus, we can utilize **W** (which must remain fixed once learned) and Eq 4 to simulate a model neuron's response to arbitrary input stimuli by replacing the column in **V** with new input. This allows us to investigate the response properties of individual model neurons much in the same way that experimental neuroscientists study biological neurons. This is important because it means that NSC can be used to model neural activity in the brain, and the resulting activity patterns generated by NSC can be compared to and evaluated against experimental findings.

It is important to note that the absence of negative weights in Eqs 2–4 does not preclude the modeling of inhibitory connections or even posit that inhibitory connections cannot participate in NSC. Rather, one important aspect of NSC is the parts-based, NMF-like decomposition of **V**; one way to achieve this is by enforcing nonnegativity constraints on **W** and **H**. Several studies have successfully incorporated inhibitory connections into their NSC-based models. One approach is to model them as nonnegative synaptic conductances. For example, Hoyer [41] used NSC to model V1 neurons as receiving input from both excitatory ON and inhibitory OFF cells in the LGN. Using prewhitened natural images, Hoyer sampled 12×12 pixel patches from the images and then separated positive and negative values into separate channels. Each image patch was thus represented by a 2×12×12 = 288 dimensional vector, each element of which mimicked the activity of an ON or OFF cell in response to the image patch. These vectors were then arranged into the columns of **V**. This procedure not only preserved the parts-based quality of the encoding but also allowed the modeling of the convergence of ON and OFF pathways. Another approach is to drop the nonnegativity constraint on **W** and thus effectively operate with both positive and negative synaptic weights. Only recently did it become clear that this approach was able to preserve the parts-based quality of the encoding (as long as nonnegativity of **H** was enforced) [44], thus simplifying the construction of more complex network topologies.

## Empirical evidence for NSC in the brain

In this section, we review evidence for NSC in several brain regions. In particular, NSC has been observed in sensory areas, an association cortex area, and the basal ganglia. Although these findings suggest that NSC might apply elsewhere in the brain, thus warranting further investigation, we are aware that NSC does not apply to everywhere in the brain. We will further discuss the limits of NSC in the Model limitations section of the Discussion.

Because of its roots in efficient coding theories of natural image processing, there is a large body of research highlighting the role of NSC in visual cortex function (e.g., [24, 35, 41, 45, 46]). More recently, NSC-like computational models have found application outside visual cortex, where they have started to provide compelling evidence that a wide variety of neuronal response properties might be understood as an epiphenomenon of efficient population coding based on dimensionality reduction. Examples include elucidating the dimensions along which perceptual space is organized in the olfactory system [47, 48], the coordination of movement in the cortico-basal ganglia-thalamo-cortical loop [3, 49], and the combined representation of allocentric and route-based spatial navigation cues in retrosplenial cortex (RSC) [50].

In the following subsections, we review studies describing evidence for NSC that either successfully explains response properties of individual neurons or has been instrumental in elucidating the dynamics at the population level. We start this section with some early modeling work that shows parts-based dimensionality reduction analogous to neuronal responses in inferotemporal cortex (IT).

### NSC in the inferotemporal cortex

The notion of parts-based object recognition is compatible with hierarchical models of vision, in which activation of simple features feeds into the activation of complex features [51]. There is a long history of debate as to whether humans detect faces based on their individual parts or as correctly arranged wholes (for reviews, see [11, 52, 53]). The working hypothesis is that the brain might use holistic face information as an early gating mechanism to allow visual stimuli access to the face processing module but that most cortical circuitry relies on parts-based information [53]. Converging evidence from human imaging studies and primate physiology suggests that faces are processed in localized “patches” within IT [54], where cells detect distinct constellations of face parts [55, 56], such as eyes [57], and that whole faces can be recognized by taking linear combinations of neuronal activity across IT [19, 58].

**Fig 4:**
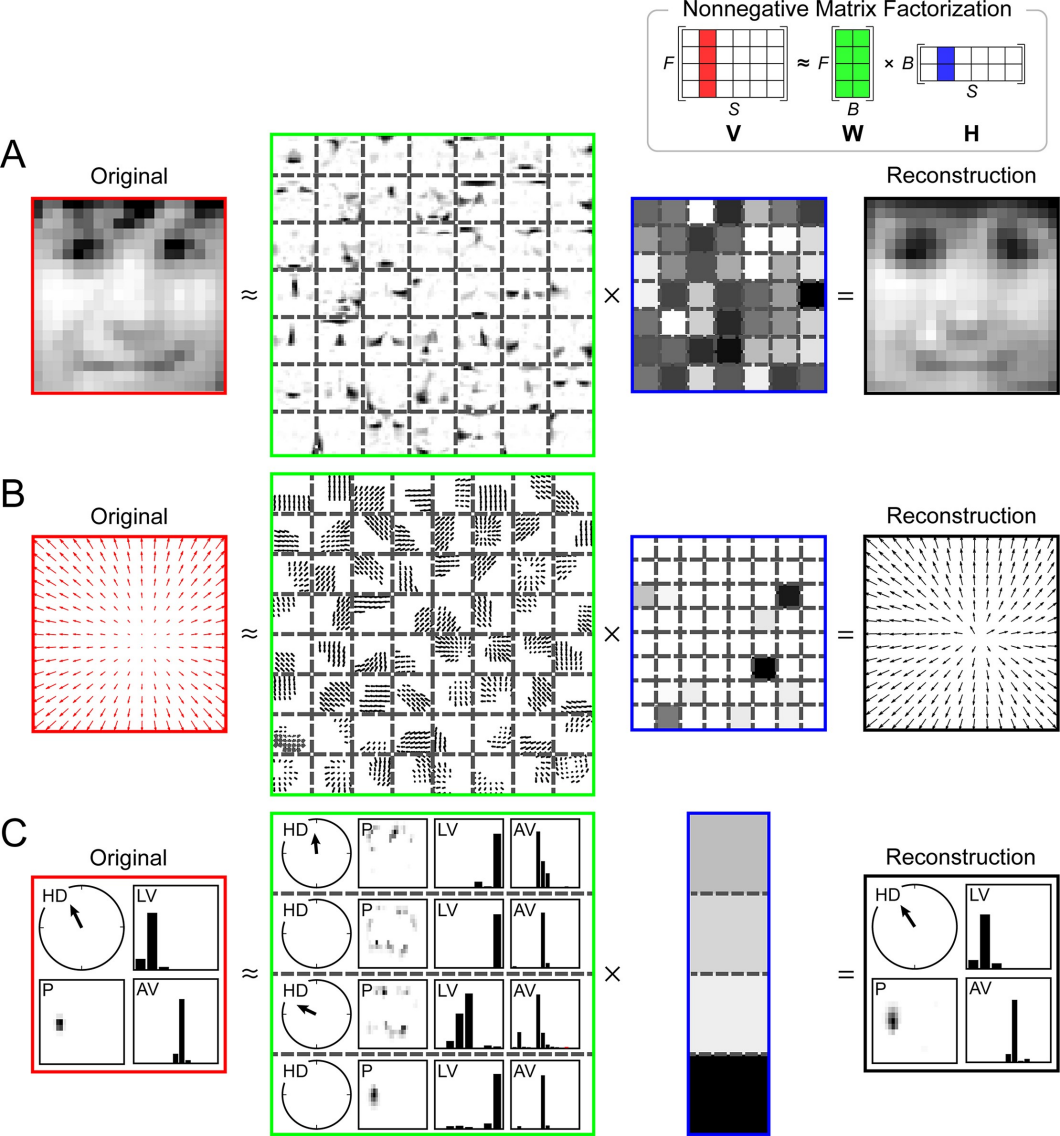
Sparse and parts-based representations recovered by NMF resemble RFs across brain regions. NMF (inset) can reconstruct a data matrix **V** (*F* features × *S* stimuli) from two reduced-rank matrices **W** (containing *B* basis functions) and **H** (containing the hidden coefficients of the decomposition). Any individual input stimulus (i.e., column in **V**, red) can be reconstructed from a linear combination (i.e., column in **H**, blue) of a set of basis functions (i.e., all columns in **W**, green). (A) A facial image can be reconstructed from a sparse activation of simulated IT neurons that preferentially respond to parts of faces (inspired by [13]). (B) An optic flow field can be reconstructed from a sparse activation of model MSTd neurons that prefer various directions of 3D self-translation and self-rotation. (C) A rat's 2D allocentric position and route-based direction of motion can be reconstructed from a sparse activation of model RSC neurons that prefer an intricate combination of LV, AV, HD, and P. For the sake of clarity, only the four most contributing hidden coefficients (out of 30) are shown. AV, angular velocity; HD, head direction; IT, inferotemporal cortex; LV, linear velocity; MSTd, dorsal subregion of the medial superior temporal area; NMF, nonnegative matrix factorization; P, 2D position; RSC, retrosplenial cortex. *Adapted with permission from [46]*.

An influential paper by Lee and Seung [13] found that applying NMF to a database of face images yielded sparse, localized features that resembled parts of a face (Fig 4A) in a similar fashion to responses in area IT. In their case, NMF acted on an *F*×*S* data matrix **V**, whose rows corresponded to distinct features of the input (e.g., *F* different pixels of an image) and whose columns corresponded to different stimuli or observations of those features (e.g., *S* different images). NMF was used to decompose the matrix into two reduced-rank matrices (Fig 4, inset) whose linear combination could be weighted such that the product of **W** and **H** provided an accurate reconstruction of **V** (see Eq 2).

A particular image, in this case encoded by *F* = 19×19 = 361 pixels could be accurately represented by a linear combination of a small number (*B* = 49) of encoding variables or “basis images” (Fig 4A). Such a representation is reminiscent to neural processing in IT, an area in the ventral visual “what” stream involved in encoding high-level object identity [58, 59], in which images of whole faces can be linearly reconstructed using responses of approximately 200 neurons that each respond to a certain set of physical facial features [19].

Interestingly, such a parts-based representation is not specific to face processing in IT; the same principle can be extended to body-selective regions in IT [60, 61].

Although there seems to be a consensus that information-theoretic explanations are relevant when investigating early sensory areas, higher-order brain areas are often considered to be specialized for performing tasks (e.g., recognizing objects, making decisions, navigating an environment) rather than the efficient encoding of information. It is therefore possible that the essential components of NSC might well be present in higher-order areas but, to date, have gone unnoticed.

### NSC in the retina

**Fig 5:**
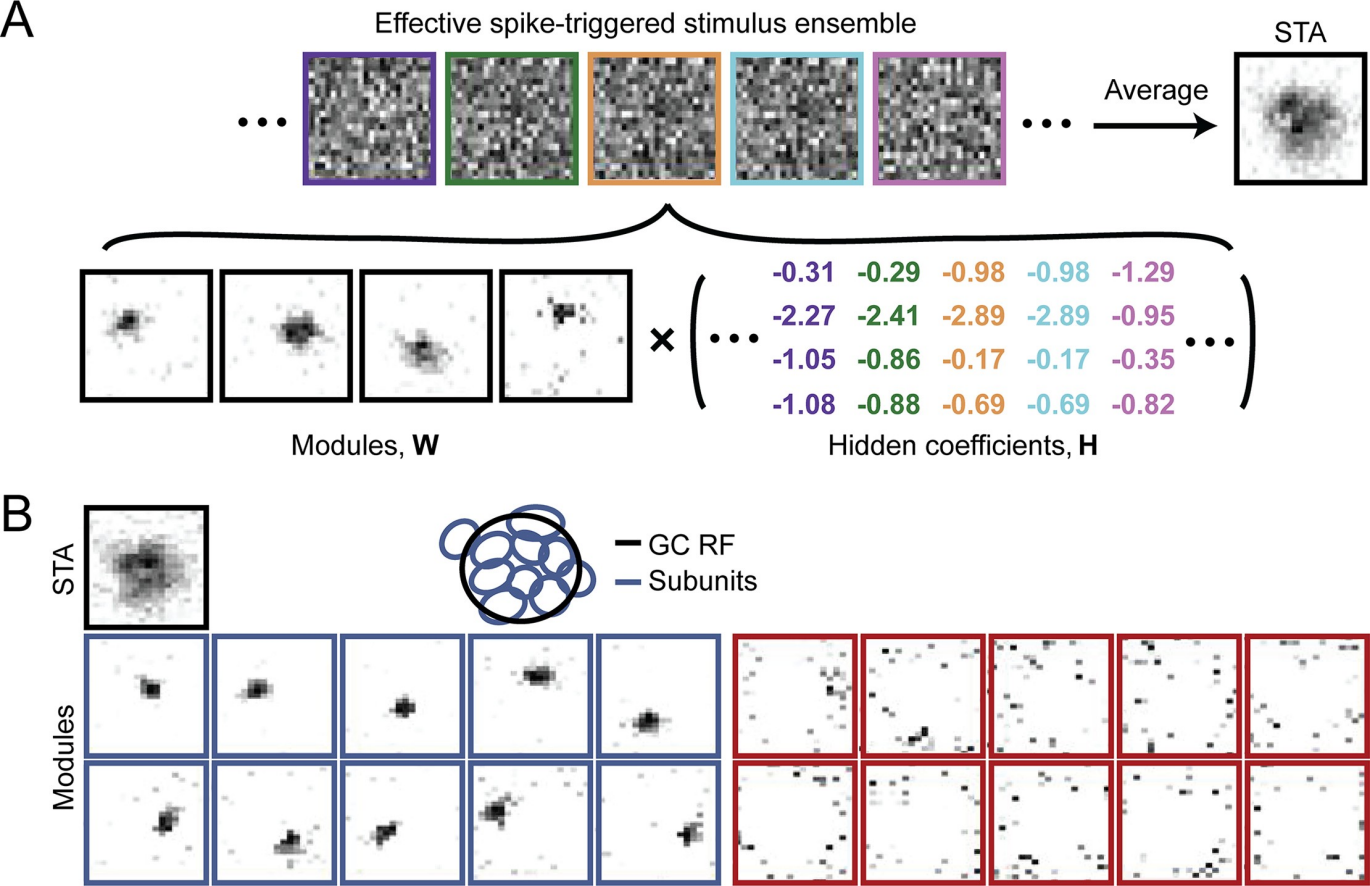
Identification of retinal ganglion cell subunits with STNMF. (A) Samples of a ganglion cell’s effective spike-triggered stimulus ensemble (top), whose average corresponds to the cell’s STA. For easier visual comparison with the subunits, STAs are displayed with negative pixel values set to zero and with zero corresponding to white in the grayscale image. STNMF decomposes this ensemble into a set of modules and hidden coefficients (bottom). The example here shows four modules that were identified for a sample ganglion cell. (B) Modules obtained for another sample ganglion cell by applying STNMF with 20 modules (bottom two rows). Some modules have a strongly localized structure (blue frames); others are more noise-like (red frames). These modules make up the subunits within a ganglion cell RF. The top row shows the cell’s RF, given by the spatial component of the STA, as well as the fitted RF outline (GC RF, black ellipse), together with outlines of the localized subunits (blue ellipses). Scale bars, 100 μm. GC, ganglion cell; RF, receptive field; STA, spike-triggered average; STNMF, spike-triggered nonnegative matrix factorization. *Adapted with permission from [44]*.

Because of its roots in efficient coding theories of natural image processing, NSC figures prominently in the vision neuroscience literature. For example, NMF-based models were able to reconstruct in vitro neuronal spike trains from the salamander retina [44, 62]. By combining spike-triggered average with NMF, Liu and colleagues [44] were able to identify the subunit layout of retinal ganglion cells (Fig 5). This technique, termed spike-triggered NMF (STNMF), involved applying NMF to the collection of those stimulus patterns contained in a spatiotemporal white-noise sequence that caused a given neuron to spike. Akin to common reverse-correlation analysis, the researchers averaged the collection of spike-eliciting stimulus segments to form the spike-triggered stimulus ensemble (Fig 5A). STNMF then decomposed the ensemble of effective spike-triggered stimuli into a matrix **W** containing a set of modules (or basis functions) and a matrix **H** containing a set of hidden coefficients.

Intuitively, the modules derived by STNMF should capture the subunit decomposition of the cell's RF because the spike-eliciting stimuli should have essential statistical structure imprinted on them by the subunits, such as correlations between pixel values [44]. And indeed, the identified modules corresponded to individual presynaptic bipolar cells, as verified by multielectrode array recordings with simultaneous recordings from individual bipolar cells through sharp microelectrodes [44]. This allowed the researchers to improve predictions about how ganglion cells respond to natural stimuli without the need to guess a specific model structure that may be constrained in terms of the size, shape, number, or nonlinearity of ganglion cell subunits.

### NSC in the early visual cortex

NSC has been extensively applied to early visual cortex, where it has successfully explained orientation and frequency tuning of simple and complex cells in V1 [41] as well as edge-like pooling of spatial frequency channels in V2 [63], including RF properties such as end-stopping and contour integration [64]. These theoretical findings are in good agreement with a large body of research documenting the sensory response of V1 across animal models (e.g., [65–68]), although they are not without controversy. For example, one study [67] criticized that some of the early sparse-coding models generated RFs that looked like stereotyped edge detectors and did not capture the diversity of RF structure observed in cat and monkey V1. However, by adjusting these models to limit the number of active neurons (“hard” sparsity) instead of limiting mean neuronal activity (“soft” sparsity), Rehn and Sommer [69] were able to account for the diversity of shapes in biological RFs. Other researchers were concerned that the apparent sparse activation of V1 was an artifact of using simple artificial stimuli such as sinusoidal gratings and drifting bars, but Vinje and Gallant [9] were able to show that natural viewing conditions actually increased the sparsity of V1 activation.

However, a number of recent studies suggest that responses are neither sparse nor low dimensional in V1 of the mouse [39, 40] and monkey [70]. Using high-density electrophysiology, Stringer and colleagues [40] found that the response of more than 10,000 visual cortical neurons to 2,000 image stimuli is high dimensional. In monkey V1, one needs to look at many principal components to decode natural images, and these principal components reflect contributions from most of the recorded neurons [70]. In addition, V1 neurons in the mouse might encode both visual stimuli and behavior in a mixed representation: a recent study found no separate sets of neurons encoding stimuli and behavioral variables, but each neuron multiplexed a unique combination of sensory and behavioral information [39]. These findings suggest that efficient coding might render an incomplete picture of sensory processing in V1 and that more studies are needed to reevaluate past findings. To this end, Stringer and colleagues [40] suggested that the population code of visual cortex might be determined by two constraints: efficiency, to make best use of the limited number of neurons, and smoothness, which allows similar stimuli to evoke similar responses.

In summary, there is a large body of research showing that computational models based on efficient coding, such as NSC, can account for a variety of response properties in early visual cortex. Although methods like spike-triggered average [71] and dimensionality reduction [72] give us confidence that we have a good understanding of the sensory response in V1, this understanding remains far from complete [73, 74] and in fact might be missing a number of dimensions related to task, state, or behavior [39, 40]. With the exception of face processing in IT [13, 19], NSC has yet to be applied to higher-order areas in the ventral visual pathway. The success of NSC in explaining V1 and V2 response properties suggests that it might be possible to extend the model to texture integration in V4.

### NSC in the dorsal visual pathway

Our group found evidence for NSC in the dorsal subregion of the medial superior temporal (MSTd) area [46], which is part of the visual motion pathway in the dorsal visual stream. Neurons in MSTd respond to relatively large and complex patterns of retinal motion (“optic flow”), owing to input from direction- and speed-selective neurons in the middle temporal (MT) area (for a recent review, see [75]). Although MSTd had long been suspected to be involved in the analysis of self-motion, the complexity of neuronal response properties has made it difficult to experimentally investigate how neurons in MSTd might perform this function.

When our group applied NMF to simulated neural activity patterns whose statistical properties resembled that of experimentally recorded MT neurons [46], we found a sparse, parts-based representation of retinal flow (Fig 4B) similar to the parts-based representation of faces encountered by Lee and Seung [13]. The resulting “basis flow fields” showed a remarkable resemblance to RFs of MSTd neurons, as they responded to an intricate mixture of 3D translational and rotational flow components in a subset of the visual field. As a result, any flow field possibly to be encountered during self-movement through a 3D environment could be represented by only *B* = 64 simulated MSTd neurons, as compared with *F* = 9,000 simulated MT input neurons. This led to a sparse and parts-based population code in which any given stimulus could be represented by only a small number of simulated MSTd neurons [46].

**Fig 6:**
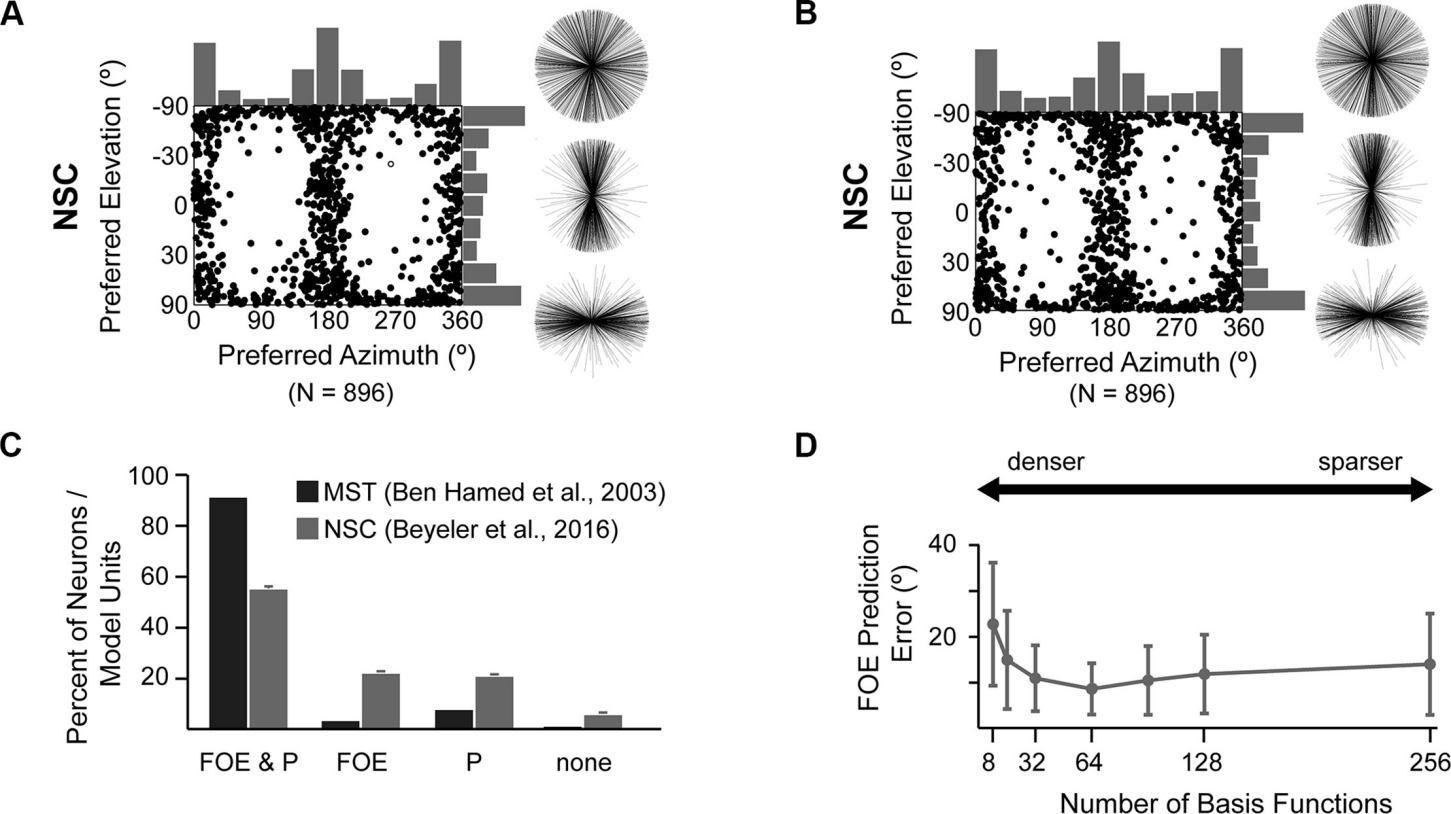
(A and B) Distribution of 3D direction preferences of MSTd-like model units in the NSC-based sparse decomposition model (rotation, [A]; translation, [B]). Each data point in the scatter plots corresponds to the preferred azimuth (abscissa) and elevation (ordinate) of a single neuron. Histograms along the top and right sides of each scatter plot show the marginal distributions. Also shown are 2D projections (front view, side view, and top view) of unit-length 3D preferred direction vectors (each radial line represents one neuron). (C) Distribution of FOE & P selectivities in macaque MSTd (dark gray) and model MSTd (light gray). Neurons or model units were involved in encoding heading (FOE), eye velocity (P), both (FOE & P), or neither (none). (D) Heading prediction (generalization) error as a function of the number of basis functions using cross validation. Vertical bars are the SD. FOE, focus of expansion; MSTd, dorsal subregion of the medial superior temporal area; NSC, nonnegative sparse coding; P, pursuit. *Reprinted with permission from [46]*.

Fig 6 shows the distribution of direction preferences of MSTd-like model units (Fig 6A and 6B; [46]) for rotation and translation, respectively. Each data point in the scatter plots specifies the preferred 3D direction of a model unit. Histograms along the boundaries show the marginal distributions of azimuth and elevation preferences. Not only did individual units match response properties of individual neurons in macaque MSTd [76]), but the model was able to recover statistical properties of the MSTd population as a whole, such as a relative overrepresentation of lateral headings.

MSTd is known to encode a number of perceptual variables, such as the direction of travel (heading) and eye rotation velocity. During forward movement, retinal flow radiates out symmetrically from a single point, the focus of expansion (FOE), from which heading can be inferred. However, instead of consisting of a set of distinct subpopulations, each specialized to encode a particular perceptual variable, MSTd has been found to consist of neurons that act more like basis functions, in which a majority of cells were involved in the simultaneous encoding of multiple perceptual variables (Fig 6C). A similar picture emerged when we investigated the involvement of MSTd-like model units in the encoding of both heading and eye rotation velocity (Fig 6C).

Interestingly, the sparsity regime in which model MSTd achieved the lowest heading prediction error (Fig 6D) was also the regime in which MSTd-like model units reproduced a variety of known MSTd visual response properties (for experimental details, refer to [46]). In contrast to findings about early visual cortex, this regime does not use an overcomplete basis set [35], yet it can still be considered a sparse coding regime [8] because only a few MSTd-like model units were needed to recover the stimulus, and each model unit responded to a subset of stimuli (see Fig 8C in [46]). Such an intermediary sparse code might be better suited (as opposed to an overcomplete basis set) for areas such as MSTd because the increased memory capacity of such a code might lead to compact and multifaceted encodings of various perceptual variables.

Taken together, the computational modeling work on MSTd described previously suggests that NSC is not specific to primary sensory areas and may be observed in other downstream sensory regions.

### NSC in the auditory cortex

Analogous to early visual cortex, the auditory system is believed to decompose auditory signals into a set of elementary acoustic features [77] such that the complete acoustic waveform can be described by a sparse population code that operates near an information-theoretic optimum [77–79]. It is therefore not surprising that computational models based on NSC have been very successful at describing the spectro-temporal RF of neurons in the primary auditory cortex (A1) [80, 81]. Response properties of A1 neurons are well described by a spectrogram; they are often tuned to stimulus frequency but are rarely phase locked to oscillations of the sound waveform [82]. The cortical representation of auditory signals seems to not only be sparse but also rely on statistically independent acoustic features [83].

Similar to visual cortex, auditory cortex is hierarchically organized, with neurons in A1 responding to simple acoustic features of natural sounds and higher-order areas responding to more behaviorally relevant stimuli. The anterior superior temporal region of auditory cortex, for example, responds to categories of acoustic objects, such as sounds produced by voices and musical instruments [82]. An intriguing question for future modeling studies is therefore whether NSC can be extended to the next level of the auditory hierarchy: Would it be possible to construct more complex acoustic objects from a sparse, parts-based set of elementary, A1-like acoustic features? And would the representation of such acoustic objects resemble neuronal responses in the anterior superior temporal region of auditory cortex?

Taken together, we suggest that auditory cortex is a good example for efficient and NSC-based coding in a sensory system other than the visual cortex, in which further study is warranted.

### NSC in the olfactory cortex

The olfactory cortex is another nonvisual cortical area worth investigating for NSC-like responses. In contrast to most other sensory modalities, the basic perceptual dimensions of olfaction remain unclear. In particular, the olfactory modality is intrinsically high dimensional and lacks a simple, externally defined basis analogous to wavelength or pitch on which elemental odor stimuli can be quantitatively compared (for a recent review, see [84]). Odors evoke complex responses in granule cells (located in the olfactory bulb) that evolve over hundreds of milliseconds [85]. Granule cells use a sparse combinatorial code to convey information about odor identity and concentration [86, 87]. Downstream from the olfactory bulb, odors tend to activate a small but consistent proportion (approximately 10%) of cortical neurons in the piriform cortex [88], which is thought to form odor object percepts [89, 90]. Although piriform cortex is not topographically organized, a spatial structure can be discerned when examining the projections of output neurons, which are highly segregated and functionally specific. Whereas the anterior piriform cortex is associated with the encoding of odor identity and odor structure, the posterior piriform cortex is involved in associational aspects of odors, such as valence and similarity [89, 91].

**Fig 7:**
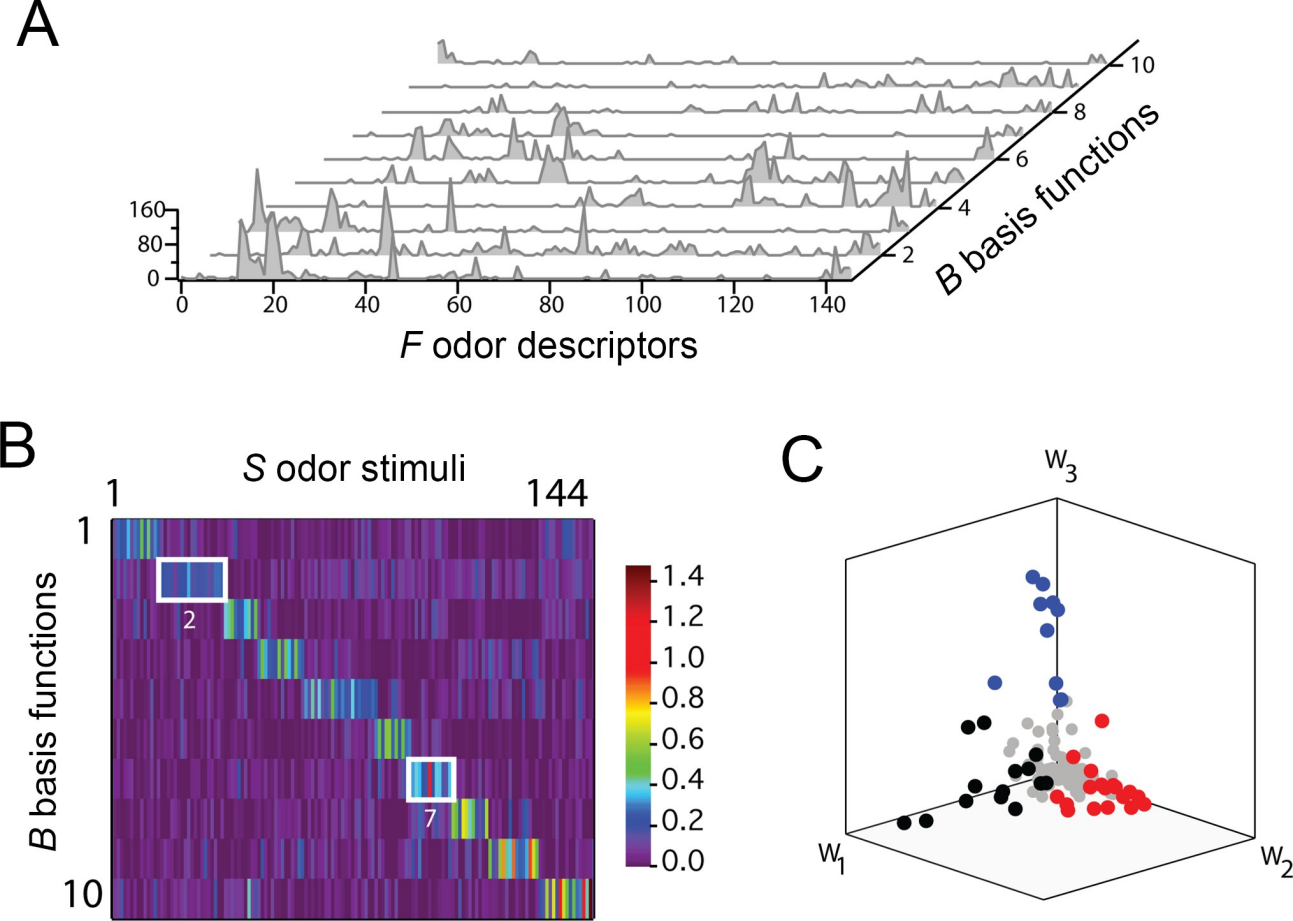
NMF recovers a sparse and parts-based representation of olfactory perceptual space. (A) Waterfall plot of the 10 basis functions constituting **W** (same nomenclature as in Fig 4). (B) Heat map of the hidden coefficient matrix, **H**, in which each column of **H** corresponds to a different odor. Columns of **H** are normalized and sorted. (C) Plot of all 144 odors in the dataset (each point is a column in **H**) in the space spanned by the first three basis functions, ${\vec{w}}_{1}$ (“fragrant”/“floral”), ${\vec{w}}_{2}$ (“woody, resinous”/“musty, earthy”), and ${\vec{w}}_{3}$ (“fruity, other than citrus”/“sweet”). Black, red, and blue points are those with their largest hidden coefficient corresponding to the first, second, and third basis function, respectively. Gray points are all remaining odors. *Adapted with permission from [48]*.

A compelling piece of evidence for NSC in the olfactory system was recently provided by Castro and colleagues [48]: In an effort to elucidate the dimensions along which perceptual space might be organized in the olfactory system, they applied NMF to a perceptual dataset built from 144 monomolecular odors, each represented by a 146-dimensional vector (an “odor profile”). Each dimension in the odor profile corresponded to the rated applicability of a number of semantic labels, such as “sweet,” “floral,” and “heavy.” By applying NMF to the odor profile, they showed that a set of 10 sparsely activated basis functions could accurately describe any odor in the dataset (Fig 7A). Interestingly, NMF revealed a prominent block diagonal structure to the full matrix **H** (Fig 7B), indicating that (1) a given odor tended to be characterized by a single prominent basis function, implying that the basis functions recovered by NMF were perceptually meaningful, and (2) all ten basis functions were being used approximately with equal frequency, implying that the basis functions recovered by NMF could span the space of behaviorally relevant odors. This suggests that a given odor percept may be considered an instance of one of several fundamental qualities.

Furthermore, NMF recovered basis functions whose descriptors aligned with perceptual dimensions highlighted in several previous analyses of odor space, including but not limited to relative pleasantness (e.g., “fragrant,” “sickening”) and potential palatability (“woody, resinous,” “chemical,” “sweet,” and “lemon”). Odors clustered predominantly along these axes (as illustrated in Fig 7C) for three specific basis functions [48].

In summary, although sensory processing in the olfactory system remains an area of active research, there is evidence consistent with a sparse and parts-based encoding of odor identity and concentration. Only recently have NSC-based methods been employed to elucidate the neural code for olfaction. Future studies may provide additional supporting evidence.

### NSC in the somatosensory cortex

In early areas of primary somatosensory cortex (S1), a number of parallels can be drawn to sparse, reduced information processing observed in other primary sensory cortices. First, activity in rodent barrel cortex, a region of S1 that is a major target for somatosensory inputs from the whiskers via the thalamus, can be extremely sparse [92–94], similar to activity in A1. Consequently, sparse-coding models have successfully explained the response properties of individual neurons in rat barrel cortex (e.g., Hafner and colleagues [95]). Second, similar to V1, neurons in primate areas 3b and 1 of S1 act like Gabor filters for tactile orientation [96, 97]. The same is true for rat barrel cortex [98]. Third, similar to visual area MT, primate S1 contains a subpopulation of neurons that can infer the direction of tactile motion from a spatiotemporal pattern of activation across a 2D sensory sheet (i.e., the skin) [99]. Specifically, neurons in area 1 of S1 tend to respond to plaid textures in the same fashion that MT neurons respond to visual plaids [99]. These findings suggest that much of what can be said about sparse and parts-based information processing in visual cortex also applies to S1.

One NSC-like model that has enjoyed success in explaining complex S1 rodent response properties is the rectified latent variable model (RLVM), a combination of nonlinear dimensionality reduction with nonnegativity constraints. In an effort to elucidate the stimulus dimensions that individual S1 neurons respond to, Whiteway and Butts [100] applied RLVM to a two-photon imaging dataset of hundreds of simultaneously recorded neurons in mouse barrel cortex while the animal was performing a tactile discrimination task. Interestingly, they found basis functions that properly identified individual neurons. Similar to the recorded neuronal responses, these basis functions were closely related to both the tactile stimulation as well as nonstimulus aspects of the behavioral task. Furthermore, RLVM achieved a lower reconstruction error than other linear dimensionality reduction techniques such as **principal component analysis (PCA)**, thus highlighting the benefit of using NMF-based decompositions over PCA to explain neural data.

However, NSC has not been observed in nonhuman primate somatosensory cortex. Tactile information from various submodalities converges at later stages of monkey S1 [101, 102] and is multiplexed across different time scales using both rate and spike timing codes [103]. These regions might represent different stages in the processing pipeline leading to form and texture perception [104]. Primate area 2 of S1 is known to integrate both tactile and proprioceptive stimuli; for example, some neurons respond only to active reaching movements, some respond only to passive movements (e.g., unexpected perturbations to the hand that generate passive limb displacements), and others respond to both [105]. These complex response properties may argue against a sparse and parts-based code in area 2.

Taken together, neurons in early somatosensory cortex respond to a small number of stimulus dimensions, not unlike to sensory neurons in early visual and auditory cortex. However, current evidence argues against NSC in higher areas of somatosensory cortex. The parallels to the visual system are striking though: area 1, which resembles visual area MT by showing Gabor-like responses to tactile motion, feeds into area 2, which resembles visual area MSTd by showing intermingling of responses to tactile and proprioceptive stimuli (analogous to intermingling of visual and vestibular stimuli in MSTd). It is therefore not unthinkable that an NSC-like model that operates on neuronal inputs to area 2—constructed analogous to [46]—could reproduce some of these response properties. However, until the neuronal mechanisms underlying these complex response properties are better understood, one would have to conclude that NSC might not apply to later stages of somatosensory cortex.

### NSC in the retrosplenial cortex

In our own work, we found evidence that NSC can explain response properties in RSC, an area important for navigation and spatial memory [106–108]. Neurons in the RSC conjunctively encode multiple variables related to the environment and one's position and movement within it (e.g., position, head direction, linear velocity, and angular velocity), allowing the representation of spatial features of the environment with respect to multiple reference frames [109].

Using a similar methodology to [46], we applied NMF, with a sparsity constraint, to parameterized behavioral variables extracted from electrophyisiological recordings of RSC neurons in the rat [109] while the animal ran back and forth on a W-shaped track (for experimental details, see Supporting information). We found a sparse and parts-based representation for behaviorally relevant variables such as the animal's position, head direction, and movement direction (Fig 4C). Interestingly, model RSC neurons encoded these variables with respect to multiple frames of reference (e.g., head direction: **allocentric reference frame**, linear velocity: **route-based reference frame**). The dimensionality of the stimulus space was drastically reduced from *F* = 417 input neurons to a set of *B* = 30 model RSC neurons.

**Fig 8:**
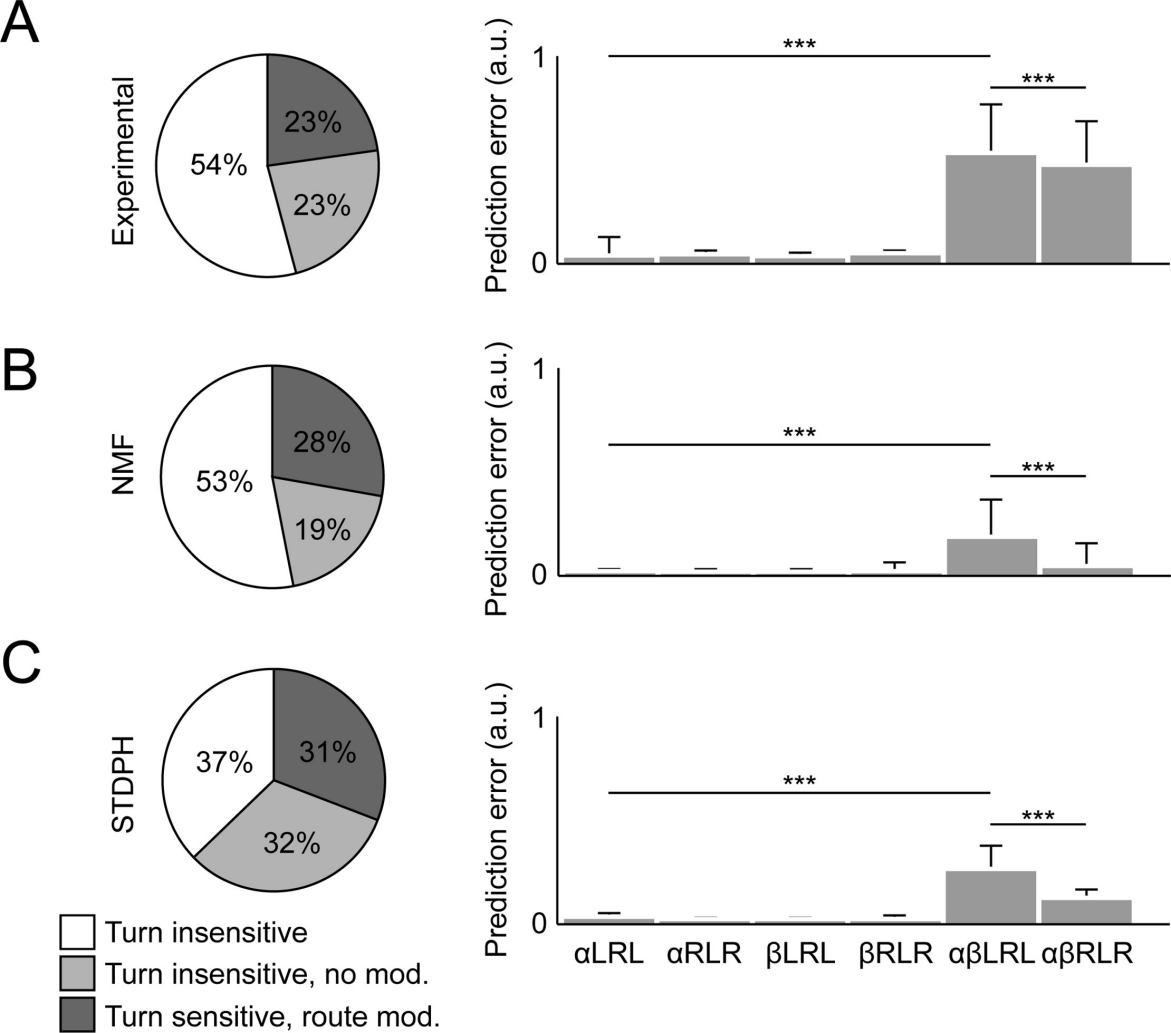
Comparison between experimental data and two computational models of rat RSC suggest a functional similarity between STDPH and NMF. Rats used two turn sequences (inbound: LRL; outbound: RLR) to traverse a W-shaped track located at two different allocentric locations (*α*, *β*). (A) Experimental data from [109]. (B) Simulated using NMF with sparsity constraints. (C) Simulated by evolving STDPH parameters to fit experimental data [127, 128]. Left column: Functional neuron type distributions. Right column: Location prediction errors. The prediction error is based on how well the neuronal population response can predict the rat's location on the maze. For details, see [50, 109]. Prediction error when comparing even and odd trials on the same maze in the same location in the room (prefix *α* or *β*) was much smaller than when the same maze was in different locations (prefix *αβ*; Kruskal-Wallis and Tukey's range test, *** = *p*<0.001), demonstrating that the network can distinguish similar routes that occur in different allocentric positions. For details see Supporting information. LRL, left-right-left; NMF, nonnegative matrix factorization; RLR, right-left-right; RSC, retrosplenial cortex; STDPH, spike-timing–dependent plasticity and homeostatic synaptic scaling.

The basis functions recovered by NMF were then used to generate simulated responses of model RSC neurons according to Eq 4, and the simulated responses were compared with neuronal responses from the electrophysiological recordings. Interestingly, simulated neuronal activity could be classified into three broad categories, with remarkably similar population statistics to rat RSC: (1) responding to left and right turns on a specific position along the route, (2) responding to left and right turns regardless of the position along the route, and (3) exhibiting complex and robust firing patterns without turn sensitivity (see Fig 8A and 8B as well as Supporting information).

Taken together, this study suggests that neuronal population activity in RSC is consistent with NSC. This is an example that NSC can apply outside sensory cortex, even where responses have not traditionally been considered sparse or parts based.

### Reinforcement-driven NSC in the basal ganglia

There is computational evidence for a reward-driven variant of NSC in the basal ganglia, a cluster of deep forebrain nuclei that are involved in the processing of motor, associative, and limbic information (for recent reviews, see [3, 110]). The basal ganglia network may be viewed as multiple parallel loops where cortical and subcortical projections interact with internal reentral loops, forming a complex network ideally designed for selecting and inhibiting simultaneously occurring events and signals (for a recent review, see [111]). To achieve this function, the basal ganglia connect most cortical areas to the frontal cortex through a series of convergent and sparsely connected pathways [112], in which signals from tens of millions of cortical neurons are projected onto a 10–10,000-fold smaller population of neurons in different subnuclei of the basal ganglia [3]. Similar to the convergence of 100 million photoreceptors onto 1 million optic nerve fibers in the retina, these highly convergent pathways from cortex to the basal ganglia suggest a potential role for dimensionality reduction.

One possible model, termed the reinforcement-driven dimensionality reduction (RDDR) model, suggests that dimensionality reduction in the cortico-basal ganglia pathway is achieved via a combination of Hebbian and anti-Hebbian learning rules that are implemented by feedforward excitatory and lateral inhibitory connections [3, 49]. These learning rules control the strength of synaptic weights in the network by altering the weight of a given synapse in proportion to the correlation between the firing rates of its presynaptic and postsynaptic neurons. In Hebbian learning, synaptic weights are strengthened given a positive correlation (leading to a phenomenon referred to as long-term potentiation [LTP]), whereas synaptic weights are depressed if the firing rate correlation is negative (leading to long-term depression [LTD]). On the other hand, in anti-Hebbian learning, which is typically applied to inhibitory connections, correlated activities are subjected to LTD, and uncorrelated activities are subjected to LTP. In order to implement dopamine-modulated Hebbian learning in this model, a reinforcement signal was used to dictate the level of dopamine in the circuit (1 for reward-related events, 0 for the absence of reward-related events, and negative values to simulate dopamine depletion) [49]. The value of the reinforcement signal then determined the sign and magnitude of each synaptic weight change.

In the RDDR model, a reinforcement signal corresponding to dopamine modulates the Hebbian learning rule of the feedforward projections, allowing the network to learn to extract input dimensions that are associated with reward activity while suppressing behaviorally irrelevant input dimensions. Whereas the original RDDR model was a neural network–based model for performing PCA [49], later iterations incorporated nonnegativity constraints on the connection weights that effectively transformed the model into an NMF variant [3]. The model predicted that these lateral connections facilitated learning by shaping correlations between neurons in the corticostriatal projections using dopamine-modulated LTP and LTD, which has yet to be experimentally validated.

In addition to suggesting a role for lateral connectivity in the basal ganglia, the RDDR model also advanced understanding of basal ganglia dysfunction in movement-related disorders such as Parkinson’s and Huntington’s disease. Previous studies had indicated that lesions to functionally healthy basal ganglia had minimal impact on behavior. Bar-Gad and colleagues [49] then suggested that this was an expected finding because of the network's ability to reorganize connections, whereas abnormal dopamine levels should significantly alter the reinforcement signal that controls the model's ability to discriminate behaviorally relevant input signals (as in Parkinson disease). Accordingly, restoration of background dopamine levels via dopamine replacement therapy alleviates the symptoms, consistent with results of dopamine depletion and restoration in the model.

In summary, NSC is a prime candidate to allow the basal ganglia to compress information in the cortico-basal ganglia pathway and extract input dimensions that are associated with reward activity. However, the complexity of the basal ganglia network has so far prohibited a deep scientific understanding of the multifaceted neural computations it performs.

## Discussion

### NSC in the brain

We reviewed compelling evidence that a wide range of neuronal responses can be understood as an emergent property of efficient coding due to dimensionality reduction and sparsity constraints. In particular, NSC might be employed by sensory areas to efficiently encode external stimulus spaces, by some associative areas to conjunctively represent multiple behaviorally relevant variables, and possibly by the basal ganglia to coordinate movement.

NSC is tightly connected to a number of unsupervised learning techniques, such as NMF (a popular tool for high-dimensional data analysis [113]), *k*-means clustering (an algorithm used to partition *n* observations into *k* clusters [114]), and **independent component analysis (ICA)** (a computational method for separating a multivariate signal into additive, statistically independent subcomponents). Both NMF and ICA are capable of decomposing high-dimensional data into parts-based representations—in contrast to PCA, which usually results in holistic representations [13]. As originally noted by Hoyer [42], if the fixed-norm constraint is placed on the rows of **H** instead of the columns of **W**, Eq 3 can be directly interpreted as the joint log-posterior of the basis functions and hidden components in the noisy ICA model [64].

Similarly, NSC is closely related to compressed sensing (for a recent review, see [115]), and a recent study has even suggested to combine the two [116]. Compressed sensing posits that neurons might implement dimensionality reduction by randomly projecting patterns of activity into a lower-dimensional space—namely, by synaptically mapping *N* upstream neurons to a downstream region containing *M*<*N* neurons. Analogously, compressed sensing supports dimensionality expansion by projecting into a larger downstream area [115]. The theory of compressed sensing then provides the mathematical tools to reconstruct the original space from the random projections.

NSC, ICA, and compressed sensing often make similar predictions that only slightly differ in the nature of the basis function representation necessary to achieve optimal reconstruction (for details, please refer to the Discussion of [115]). For example, whereas ICA emphasizes the statistical independence of unmixed sources, and compressed sensing requires basis function to be “maximally incoherent”[115], NSC does not make any such assumptions as long as the basis functions are nonnegative.

#### Potential neural mechanisms

To operate efficiently, it has been suggested that the brain might enforce geometrical and biophysical constraints on axonal wiring [117]. In addition to reducing overall wiring length [118], the brain might also aim to minimize local delays by favoring a high degree of local connectivity [119]. If connectivity reflects coding [120], it would not be surprising to find that such ecological considerations carry over into brain function, favoring sparse population codes and neuronal representations that are local in functional space (i.e., parts-based). However, wiring cost is likely to be only one of many constraints on the brain connectome, perhaps supplementing competitive pressures for hub-mediated information exchange between network modules [121].

In addition, evidence suggests that Hebbian-like synaptic plasticity rules allow neurons to perform statistical inference on their inputs [47, 122–124]. One particular study demonstrated through a mathematical proof that a certain form of **spike-timing–dependent plasticity (STDP)** in combination with homeostatic synaptic scaling (i.e., STDP and homeostatic synaptic scaling [STDPH]) can approximate the NMF algorithm [123]. Similar to Oja's rule [124], which was developed to stabilize rate-based Hebbian learning (effectively resulting in PCA), Carlson and colleagues showed that synaptic scaling acts as a homeostatic mechanism to stabilize STDP (effectively resulting in NMF). Interestingly, we were able to apply these ideas to electrophysiologically recorded neuronal activity observed in the RSC of rats during a spatial navigation task (Fig 8; for experimental details, see Supporting information). Both STDPH and NMF were able to recover key features such as encoding spatial reference frames (i.e., allocentric and route-centric firing patterns) and position discrimination by reducing the dimensionality of behavioral variables (e.g., velocity, head direction, position). The neuronal and population responses from NMF and STDPH were comparable to the experimental findings [109]. Furthermore, the STDPH model contained a highly flexible and generalizable code that could automatically encode new routes through the same environment without retraining [50].

However, more research is needed to elucidate any potential connection between NSC and the many different synaptic plasticity rules commonly found across brain regions, different stages in the life of an animal, and animal species (e.g., [125, 126]).

#### Model limitations

Although NSC has proved useful in understanding a variety of neuronal responses as an emergent property of efficient population coding based on dimensionality reduction and sparse coding, it is clear that it does not apply to everywhere in the brain. In this section, we discuss some of the limitations to this theory.

First, there is increasing evidence that several motor variables are encoded throughout the brain, including early sensory areas [39, 129–131]. For example, in the mouse, running modulates the gain of visual inputs [132, 133] and is critical for integration of visual motion [134, 135]. A potential explanation for these widespread effects is that certain movements reflect changes in the animal's internal state, such as increased arousal during running [133]. Interestingly, these results were not specific to visual cortex but were seen across wide regions of the mouse forebrain, suggesting that neuronal activity in these areas is more than just an efficient encoding of sensory stimuli.

Second, a practical limitation of dimensionality analyses in general is that the apparent dimensionality of the population response changes systematically with the complexity of the input space [8, 72, 136]. For practical purposes, simulated models of neuronal circuitry are typically built with far fewer units than the number of neurons in the real network. By excluding input dimensions that are present in the brain, one will implicitly guide the simulated model away from spurious interactions that the real circuitry would have to handle. As a result, the simulated model might underestimate the true complexity of the task. Rather than trying to put an absolute number to the dimensionality of neuronal population activity in a given brain area, one should instead systematically vary the input to said brain area and ask whether the outputs of dimensionality reduction change in a sensible manner [72].

Third, part of the confusion about the role of sparse coding in the brain may arise from the wide variety of definitions of sparsity used in the literature [8, 38]: sparse population activity does not necessarily imply a sparse-coding scheme. In its widest possible theoretical sense, a neuron population exhibits sparse activity if the average activation ratio remains below 50% for binary neurons or below 100% for thresholded, rate-based neurons [8]. However, it is not surprising that different brain areas might employ different degrees of sparsity. For example, a network of “grandmother” cells might be able to implement a maximally sparse code, but it would have to do so by sacrificing representational capacity (as in Fig 1) and fault tolerance (i.e., the capacity to handle neuronal noise or the loss of a subset of neurons) [5, 8]. Instead, some brain areas might prefer to operate at a degree of sparsity that still allows for some level of robustness or adaptability. It is conceivable that this “point of operation” might depend on the complexity of the stimulus to be encoded or the task to be performed—for example, favoring an extremely sparse code in V1 [35] but giving rise to a slightly denser code with greater representational capacity in higher-order visual areas such as MSTd, which could lead to compact and multifaceted encodings of various perceptual variables (see Discussion in [46]).

Furthermore, NSC does not apply to many regions of the brain, especially when the role of the brain area is to cause behavior. Two prominent examples in which NSC has not been observed are the prefrontal cortex (PFC) and motor cortex. For one, studies indicate that the population code in these regions may be quite dense (as in Fig 1A). For another, dimensionality reduction studies in these regions suggest that individually neurons typically encode multiple task-related signals at once, such as the animal's upcoming choice, state, and the strength of sensory evidence [14, 15, 137–140]. Whereas feedforward neural networks are better predictors of neuronal processing in early sensory areas, it is interesting to note that motor activity seems to be well represented by recurrent neural networks [141] as well as models based on random projection theory [142].

There is increasing evidence that responses in the primary motor cortex (M1) are neither sparse nor parts based [137, 138, 143–146]. Instead, M1 neurons tend to be active during most movements [145], and response patterns of individual neurons rarely match those of individual muscles [138, 144]. In addition, M1 neurons show temporally complex patterns of activity leading to high-dimensional population activity [138], as measured by PCA during a reaching task. These findings might be due to the role of the motor system, which is to cause behavior rather than represent features. Motor cortex can be understood as part of a larger dynamical system spanning many areas, including the spinal cord, and incorporating sensory feedback (for a recent review, see [146]). In this view, correlations between activity and movement parameters need not represent anything so long as the right patterns of activity are created at the level of the spinal cord [147–149].

### Potential for NSC in other brain regions

There are several nonsensory areas that may demonstrate NSC. In this section we point to evidence that suggests this is the case but also discuss how sparse activity in these regions differs from NSC in sensory systems. We suggest that further studies should be carried out to assess the potential for NSC in these regions.

#### Hippocampus

There are aspects of the hippocampus that are comparable to the ideas of NSC proposed here. For example, the dentate gyrus is often associated with sparse activity [150–152]. The expansion from a dense, enthorhinal cortex coding to sparse dentate gyrus activity suggested a mechanism of pattern separation [153, 154]. Since these early theories, accumulating evidence has supported the role of the dentate gyrus in pattern separation [150, 152, 155]. However, the dentate gyrus projects to a highly recurrent CA3 region, which does not appear to be sparse or reduce dimensionality. Rather, the CA3 region has a role in pattern completion through autoassociation [153, 154, 156]. Another feature of hippocampal processing that is related to NSC may be the place cell itself. Sparsity has long been used as a metric for place cell quality [157]. A place cell is said to be sparse if it fires in a small region of the environment, and this is related to how much spatial information that cell encodes. In a sense, each place cell can be thought of as a "part" of the environment, and these parts cover the entire environment. Although this evidence points to sparse, parts-based, and reduced coding in some hippocampal regions, it does differ from the NSC representations in sensory areas. That is, sparse coding as defined previously for sensory areas is not necessarily the same as sparsity or as sparse activity leading to pattern separation. Still, it would be of interest to apply NSC to the hippocampal inputs, similar to the methods applied to RSC during a spatial navigation task [50], to see if sparse, reduced basis functions emerge in other regions.

#### Parietal cortex

Analogous to our modeling work in MSTd [46], it might be possible to apply NSC to other areas of the posterior parietal cortex that are involved in multisensory heading perception. Areas such as the ventral intraparietal (VIP) area and the visual posterior sylvian (VPS) area are also known to respond to optic flow, but they increasingly respond to inertial vestibular stimulation as well [158]. Although the degree of sparsity of the population code in VIP and VPS is not known, the fact that neurons in these areas respond to mixtures of visual and vestibular heading cues make them prime examples to be examined with an NSC-based modeling approach.

Elsewhere in parietal cortex, single neurons act as basis functions to represent the spatial configuration of objects with respect to multiple reference frames (e.g., by transforming eye-centered to body-centered coordinates) [18, 159, 160]. This is similar to the integration of multimodal heading cues mentioned previously, as well as to other associative areas such as RSC, which demonstrates conjunctive coding of various spatial navigation cues [50, 109]. There is further evidence that actions are represented in parietal cortex with respect to arbitrary and abstract reference frames, such as with respect to a planned route through an environment [161]. From a theoretical standpoint, NSC seems a good candidate to find an efficient, reference frame–agnostic representation of various behaviorally relevant variables [27, 45, 162, 163], but future studies will have to address these issues step by step.

### Future directions

In addition to the areas highlighted previously, the essential components of NSC might be present in other brain regions not traditionally associated with the efficient encoding of information. We offer three testable predictions of this theory:

First, we suggest that a variety of neuronal response properties can be understood as an emergent property of efficient population coding based on dimensionality reduction. Depending on input stimulus and task complexity, we expect the dimensionality of the population code to be adjusted according to the bias–variance dilemma (Fig 3). This point of operation might differ across brain areas—for example, favoring neurons that respond to a small number of stimulus dimensions in V1 [35] but giving rise to mixed selectivity in higher-order brain areas such as MSTd [46] and RSC [50, 164].

Second, we predict that parts-based representations can explain RFs of neurons in a variety of sensory and associative cortices, including but not limited to those brain areas discussed here. In agreement with the literature on basis function representations [18, 159, 160], we expect parts-based representations to be prevalent in regions where neurons exhibit a range of tuning behaviors [46], display mixed selectivity [165, 166], or encode information in multiple reference frames [50, 109, 164].

Third, where such representations occur, we expect the resulting neuronal population activity to be relatively sparse in order to encode information both accurately and efficiently. As noted previously, sparse codes offer a trade-off between dense codes (in which every neuron is involved in every context, leading to great memory capacity but suffering from cross talk among neurons) and local codes (in which there is no interference but also no capacity for generalization).

## Conclusion

In conclusion, there is increasing evidence that NSC can account for neuronal response properties in a number of sensory and associative cortices, as well as subcortical areas such as the basal ganglia. Although NSC might not apply to all brain areas—for example, motor or executive function areas—the success of NSC-based models, especially in sensory areas, warrants further investigation for neural correlates in other regions.

## Data availability

The software used to generate some of the data presented in Figs 1B, 2 and 6A is archived on Zenodo (10.5281/zenodo.2641351). The latest version is available on GitHub: https://github.com/mbeyeler/2019-nonnegative-sparse-coding.

## Glossary

- **Allocentric reference frame.** A spatial frame of reference that is defined with respect to a broader environment (e.g., one's location on a map). Hippocampal place cells are a textbook example of neurons that are anchored to an allocentric reference frame
- **Basis functions.** A lower-dimensional set of linearly independent elements that can represent a high-dimensional input space given a weighted sum of these elements, in which the weight of each element is defined by a separate hidden component. For example, according to Fourier analysis, sine and cosine are basis functions for the space of all continuous periodic functions.
- **Dimensionality reduction.** The process of reducing the dimensionality of a space to the lowest possible space that encapsulates the variance of the original data via feature extraction. In the case of neuronal firing rate patterns, this means representing all possible firing rate patterns in the brain region using the smallest possible subset of the neurons
- **Efficient coding.** A theoretical model of sensory coding in the brain based on information theory [24–26]. The efficient coding hypothesis posits that sensory pathways can be understood as communication channels in which neuronal spiking aims to maximize available channel capacity by minimizing the redundancy between representational units
- **Holistic representation.** Representation of a stimulus space that does not rely on explicit representations of stimulus component parts. For example, a house might be represented by the visual system as a set of house “templates.” Although visual information from individual house components (e.g., front door, windows, roof, etc.) would of course be included in the house representation, that information would be not be contained in representational packets corresponding to the parse of the house into these features. Instead, houses would be recognized “all of a piece.
- **ICA.** A computational method for decomposing multivariate data into additive components by assuming that the components are non-Gaussian signals and statistically independent from each other. Independent components differ from decorrelated components by the fact that the minimization includes higher-order and not only second-order statistics. A simple application of ICA is the “cocktail party problem,” in which the underlying speech signals are separated from a sample data consisting of people talking simultaneously in a room
- **NMF.** A computational method for decomposing multivariate data into additive components by constraining the components to be nonnegative. This constraint results in a parts-based representation because it only allows additive and not subtractive combinations of subcomponents
- **Parts-based representation.** Representation of a stimulus space in terms of explicit representations of stimulus component parts. For example, a house might be decomposed by the visual system into a set of doors, windows, a roof, etc. The resulting representation of the house would consist of representations of these parts, somehow linked together
- **PCA.** A computational method for decomposing multivariate data into linearly uncorrelated components. PCA identifies an ordered set of orthogonal directions that captures the greatest variance in the data [14]
- **Representational capacity.** The number of recognizably different patterns of neuronal activity that a population of neurons can generate in a useful time interval [167]
- **Route-centric reference frame.** A spatial frame of reference that is defined with respect to a planned path through a broader environment. For example, neurons in some parts of the brain fire for a particular location in a route, even if the route is repositioned or reoriented in the broader environment [161]
- **Sparse coding.** A population coding scheme in which activity is represented by the strong activation of a relatively small set of neurons. Sparse coding can be described as a trade-off between the benefits and drawbacks of dense and local codes [5]
- **STDP.** A Hebbian-inspired learning rule in which weight updates are computed based on the precise spike times of pre- and postsynaptic neurons that induce either LTP or LTD in the synapse, depending on whether the total presynaptic spike count preceded the total postsynaptic spike count, integrated over a critical temporal window

## Supporting information

S1 Supplemental Information Experimental details: Dimensionality reduction in RSC. RSC, retrosplenial cortex.
(PDF)

S1 Fig SNN architecture used in the RSC experiment. The SNN had four input groups and a total of 417 excitatory input neurons (390 neurons for position, eight for head direction, 12 for linear velocity, and seven for angular velocity). There were a total of 600 output Izhikevich neurons, of which 80% (480) were excitatory neurons and 20% (120) were inhibitory neurons. Network connectivity was set at 10% probability across all connections (inp → inh, inp → exc, inh → exc, and exc ↔ exc), and each connection type was governed by its own STDPH curve (excitatory STDP on the inp → exc, inh → exc, and exc ↔ exc connections and inhibitory STDP on the inp → inh connections). exc, excitatory; inh, inhibitory; inp: input; RSC, retrosplenial cortex; SNN, spiking neural network; STDPH, spike-timing–dependent plasticity with homeostatic synaptic scaling.
(PDF)
