# Supplementary material for: Neural correlates of sparse coding and dimensionality reduction
Source: PLoS Comput Biol. 2019 Jun 27;15(6):e1006908. doi: 10.1371/journal.pcbi.1006908 (PMC6597036; doi:10.1371/journal.pcbi.1006908)
Supplement: S1 Supplemental Information — RSC, retrosplenial cortex. (PDF) [file pcbi.1006908.s001.pdf]

Neural correlates of sparse coding and dimensionality reduction

Michael Beyeler[1,2,3,4,☯,*], Emily L. Rounds[5,☯], Kristofor D. Carlson[5,6], Nikil Dutt[4,5], Jeffrey L. Krichmar[4,5]

1 Department of Psychology, University of Washington, Seattle, WA, USA
2 Institute for Neuroengineering, University of Washington, Seattle, WA, USA
3 eScience Institute, University of Washington, Seattle, WA, USA
4 Department of Computer Science, University of California, Irvine, CA, USA
5 Department of Cognitive Sciences, University of California, Irvine, CA, USA
6 Sandia National Laboratories, Albuquerque, NM, USA

☯These authors contributed equally to this work.
* Corresponding author. Email: mbeyeler@uw.edu

Supplemental Information

Experimental details: Dimensionality reduction in retrosplenial cortex (RSC)

Alexander and Nitz [1] investigated the spatial reference frame(s) to which retrosplenial cortical activity is anchored. In their experiments, six Long-Evans rats traversed a W-shaped track that occupied two distinct positions in the recording room, referred to as track positions α and β, which varied by recording session. The track was composed of two sets of action sequences depending on the direction of traversal (outbound or inbound trials). Outbound and inbound runs were made up of opposite turn sequences (left-right-left (LRL) and right-left-right (RLR), respectively. The manipulation of turn sequence, route, and track position allowed the assessment of neural sensitivity to the *allocentric*, *egocentric* and *route-based* reference frames by comparing the observed firing patterns of electrophysiologically recorded neurons by the rat's position with respect to the route, the room, and the action being performed.

In total, 243 neurons were recorded over 71 recording sessions, with measures taken to ensure maximum independence between the neurons that were recorded in each session. Each session had approximately 20 trials per condition (track position by direction of traversal). Additionally, Alexander and Nitz [1] recorded relevant behavioral data concurrently with neuronal firing patterns for each trial, including head direction (HD), position in X-Y coordinates (Pos), linear velocity (LV), and angular velocity (AV).

Our group sought to develop computational models that could replicate the functional, behavioral, and population responses observed in the electrophysiologically recorded data in response to the recorded behavioral metrics HD, Pos, LV, and AV [2,3]. We considered two different approaches:

- Similar to [4], we applied nonnegative matrix factorization (NMF), with a sparsity constraint, to the parameterized behavioral variables that were used as input to the network in order to generate another set of synthetic neuron responses. The functional properties of the synthetic neurons were compared to the electrophysiological dataset.

- We used an evolutionary strategy to evolve parameters corresponding to spike-timing dependent plasticity and homeostatic synaptic scaling (STDPH) in a population of spiking neural networks (SNNs) that could replicate the electrophysiological dataset [2, 3].

Methods

Applying NMF with sparsity constraints

Similar to [4], we used the idealized neural activity to construct a matrix of training data associated with each of the four recorded 'features' (i.e., linear velocity, angular velocity, head direction, and position). Using Gaussian and cosine tuning curves, we created $F = 417$ idealized input neurons that encoded these four variables, and arranged them into a data matrix $\mathbf{V}$, to which NMF was applied.

We tested a range of values for the number of basis functions B to which the input matrix would be reduced; ultimately, 30 basis functions best captured the results of the experimental data.

Fitting STDPH parameters in a spiking network

In addition, we used an evolutionary strategy to evolve parameters corresponding to STDPH in a population of SNNs that could replicate the functional, behavioral, and population responses observed in the electrophysiologically recorded data in response to the recorded behavioral metrics HD, Pos, LV, and AV [2,3]. Simulations were performed using CARLsim [5].

The network architecture is shown in Fig. S1. Each network contained 600 neurons (480 excitatory and 120 inhibitory Izhikevich neurons [6]). Each trial consisted of 200 bins, each associated with a specific combination of these four inputs. The recorded values were encoded using cosine and Gaussian tuning curves that were subjected to a Poisson process to produce spiking inputs. The population was allowed to evolve over 50 generations (convergence was observed to occur by approximately the 20th generation).

Synthetic neural activity was averaged across trials for each track position/traversal combination and then correlated with the 243 electrophysiologically recorded neuronal firing patterns. For each of the electrophysiologically recorded neurons, the synthetic neuron with the highest-correlated firing pattern was assigned as a match for that neuron. No duplicate matches were allowed—a neuron could be matched only once.

Each SNN in the population was evaluated according to a fitness function that measured the sum of the highest correlations between neurons, with a penalty for overly high average maximum firing rates for the synthetic neurons to ensure a stable firing regime. The networks consistently converged to a fitness value of 105.93 ± 0.91 (arbitrary units), or an average correlation of Pearson's $R = 0.43$ per neuron (high correlations by experimental standards).

Results

We found that the activity patterns of both NMF and STDPH model neurons could replicate the neuronal response properties and population activity seen in the electrophysiologically recorded neurons in the dataset (see main text); that is, the model neuron activity could be classified into three broad categories, with remarkably similar population statistics to rat retrosplenial cortex (RSC) [1]:

- Turn-sensitive, no modulation neurons responded whenever the animal made a left or right turn on the track (light gray);

- Turn-sensitive, route modulation neurons responded whenever the animal made a turn on a specific position along the route, independent of allocentric location (dark gray); and
- Turn-insensitive neurons that did not reflect turning behaviors or actions performed by the rats, but nonetheless exhibited complex and robust firing patterns (white). This set of neurons appear to encode information related to position in allocentric space with modulation by other variables.

In addition, both NMF with sparsity constraints and STDPH model RSC produced simulated neurons whose combined population activity could be used to predict the agent's location on a route with respect to the allocentric frame of reference. Population activity patterns could also disambiguate the agent's location within routes that occupied different locations in the room, consistent with findings of population behavior in the biological RSC [1].

When even and odd trials on the same track locations were compared, prediction error was very low, but when the tracks were in two different locations (i.e., α vs. β), the prediction error was significantly higher in all cases (see Fig. 8A, B, C, right in the main text).

For further details on this analysis, please refer to [1–3].

References

1. Alexander AS, Nitz DA. Retrosplenial cortex maps the conjunction of internal and external spaces. Nature Neuroscience. 2015;18:1143–1151. doi:10.1038/nn.4058.

2. Rounds EL, Scott EO, Alexander AS, DeJong KA, Nitz DA, Krichmar JL. An Evolutionary Framework for Replicating Neurophysiological Data with Spiking Neural Networks. In: Handl J, Hart E, Lewis PR, López-Ibáñez M, Ochoa G, Paechter B, editors. Parallel Problem Solving from Nature – PPSN XIV: 14th International Conference, Edinburgh, UK, September 17-21, 2016, Proceedings. Springer International Publishing; 2016. p. 537–547.

3. Rounds EL, Alexander AS, Nitz DA, Krichmar JL. Conjunctive Coding in an Evolved Spiking Model of Retrosplenial Cortex. Behavioral Neuroscience. 2018;132(5):430–452.

4. Beyeler M, Dutt N, Krichmar JL. 3D Visual Response Properties of MSTd Emerge from an Efficient, Sparse Population Code. The Journal of Neuroscience. 2016;36(32):8399–8415. doi:10.1523/JNEUROSCI.0396-16.2016.

5. Beyeler M, Carlson KD, Chou TS, Dutt ND, Krichmar JL. CARLsim 3: A user-friendly and highly optimized library for the creation of neurobiologically detailed spiking neural networks. In: Neural Networks (IJCNN), 2015 International Joint Conference on; 2015. p. 1–8.

6. Izhikevich EM. Simple model of spiking neurons. IEEE Transactions on neural networks. 2003;14(6):1569–1572.
