# Supplementary material for: Neural correlates of sparse coding and dimensionality reduction
Source: PLoS Comput Biol. 2019 Jun 27;15(6):e1006908. doi: 10.1371/journal.pcbi.1006908 (PMC6597036; doi:10.1371/journal.pcbi.1006908)
Supplement: S1 Fig — The SNN had four input groups and a total of 417 excitatory input neurons (390 neurons for position, eight for head direction, 12 for linear velocity, and seven for angular velocity). There were a total of 600 output Izhikevich neurons, of which 80% (480) were excitatory neurons and 20% (120) were inhibitory neurons. Network connectivity was set at 10% probability across all connections (inp → inh, inp → exc, inh → exc, and exc ↔ exc), and each connection type was governed by its own STDPH curve (excitatory STDP on the inp → exc, inh → exc, and exc ↔ exc connections and inhibitory STDP on the inp → inh connections). exc, excitatory; inh, inhibitory; inp: input; RSC, retrosplenial cortex; SNN, spiking neural network; STDPH, spike-timing–dependent plasticity with homeostatic synaptic scaling. (PDF) [file pcbi.1006908.s002.pdf]

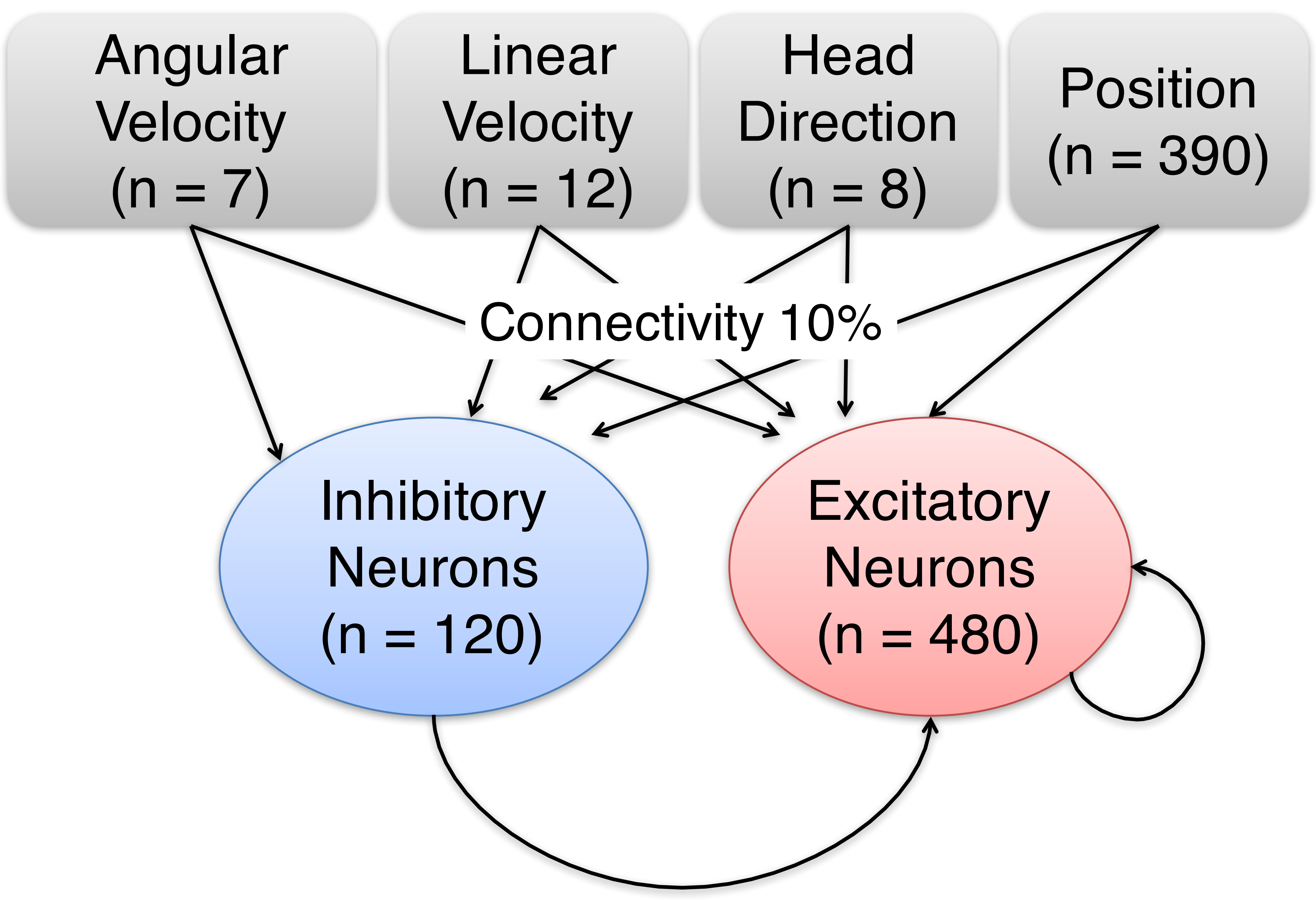
Angular Velocity (n = 7)
Linear Velocity (n = 12)
Head Direction (n = 8)
Position (n = 390)
Connectivity 10%
Inhibitory Neurons (n = 120)
Excitatory Neurons (n = 480)
